# Triol-promoted activation of C–F bonds: Amination of benzylic fluorides under highly concentrated conditions mediated by 1,1,1-tris(hydroxymethyl)propane

**DOI:** 10.3762/bjoc.9.283

**Published:** 2013-11-13

**Authors:** Pier Alexandre Champagne, Alexandre Saint-Martin, Mélina Drouin, Jean-François Paquin

**Affiliations:** 1 Canada Research Chair in Organic and Medicinal Chemistry, CCVC, PROTEO, Département de chimie, Université Laval, 1045 avenue de la Médecine, Québec, QC, Canada G1V 0A6

**Keywords:** C–F bond activation, highly concentrated conditions, nucleophilic substitution, hydrogen bond, organofluorine, triol

## Abstract

Activation of the C–F bond of benzylic fluorides was achieved using 1,1,1-tris(hydroxymethyl)propane (**2**) as a hydrogen bond-donating agent. Investigations demonstrated that hydrogen bond-donating solvents are promoting the activation and hydrogen bond-accepting ones are hindering it. However, the reaction is best run under highly concentrated conditions, where solvents cannot interfere with the interaction between the organofluorine compound and the triol. Various benzylic fluorides react with secondary amines or anilines to form benzylic amines in good yields.

## Introduction

**Figure 1:**
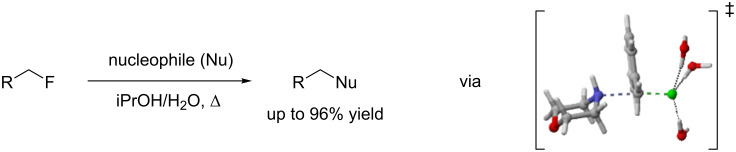
S_N_2 reaction of activated alkyl fluorides and calculated transition state for the reaction of morpholine with benzyl fluoride with three molecules of water.

The discovery of mild methods for the activation of C–F bonds is of high importance both from a fundamental point of view as well as for potential practical applications [1]. Specifically for aliphatic monofluorides, a number of transition metal-catalyzed methods [1–9] and transition-metal-free methodologies [1,10–12] have been developed. In continuation with our interest in the activation of C–F bonds [13–16], we have recently reported that it was possible to enable the use of fluoride as a leaving group in nucleophilic substitution reactions of activated alkyl fluorides through hydrogen bonding [17]. Particularly, water was used as the hydrogen bond donor and co-solvent. DFT calculations show that activation proceeds through stabilization of the transition-state structure by, amongst other things, hydrogen bonds between the fluorine atom and the water molecules, and not simple transition-state electrostatic stabilization by the solvent even though a mixture of polar solvent (iPrOH/H_2_O in a 1:1 ratio) is used (Figure 1).

**Figure 2:**
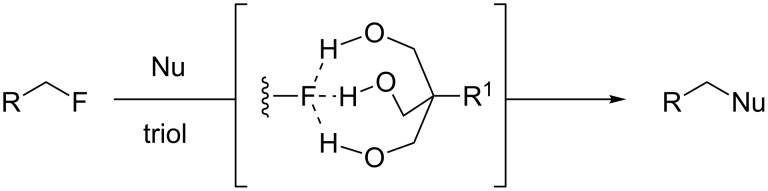
Proposed activation of C–F bonds mediated by a triol.

For its activating role in our system, water appeared to work mostly as a triad of spatially and geometrically well-defined hydrogen bond-donating moieties. We therefore wondered about what would happen if these three moieties (water molecules) were covalently linked together in the form of a triol, which could help the three O–H functionalities to position themselves strategically around the three lone pairs of fluorine, which acts as a hydrogen bond acceptor [18–21] (Figure 2). Revoking the need to have five molecules (substrate, nucleophile and three water molecules) in a precise geometry could also enable a faster reaction with less activating agent, which means we could potentially use the triol as an additive rather than a solvent. Altogether, this strategy would represent a unique metal-free and unprecedented small-molecule-mediated activation of C–F bonds.

Herein, we report the feasibility of this concept for the amination of benzylic fluorides, activated alkyl fluorides [22], using 1,1,1-tris(hydroxymethyl)propane as the triol. Furthermore, optimization of the reaction conditions have revealed that the reaction was best run under highly concentrated conditions [23].

## Results and Discussion

Our initial investigations were performed using 4-phenylbenzyl fluoride (**1**) [24] as the substrate and morpholine (3 equiv) as the nucleophile at 60 °C for 24 h. Commercially available 1,1,1-tris(hydroxymethyl)propane (**2**) was selected as the triol and was used in a slight excess (1.1 equiv) [25] relative to the substrate. Solvent screening was completed with and without added triol **2** in order to establish its effect on the reactivity and results are reported in Table 1. In water or alcoholic solvent without added triol, low to moderate conversions were observed (12–26%). This was expected since these are all hydrogen bond-donor solvents [17,26]. A low but quantifiable increase in conversion is observed when **2** is added to these reactions, with the best result (35%) being obtained in water (Table 1, entries 1–3). While the effect of the triol seems minimal, it is possible in these cases that **2**, being both a hydrogen bond donor and acceptor, is engaged in a hydrogen bond network with the solvent, thus limiting its availability for the benzylic fluoride. Interestingly, switching to solvents with better acceptor than donor properties impeded the reaction [26]. Hence, the use of toluene (Table 1, entry 4), EtOAc (Table 1, entry 5), THF (Table 1, entry 6) and DMF (Table 1, entry 7) provided at best traces of the desired benzylic amine **3**. We speculate that the ability of the solvent to interact more strongly with the triol than would the substrate results in no reaction. Finally, "inert" solvents [26–27] such as hexane (Table 1, entry 8) and CH_2_Cl_2_ (Table 1, entry 9) were investigated. In both cases, little reaction was observed without **2**, and moderate conversions were obtained with **2**. In this instance, the solvent is unable to interact with either the triol or the substrate and the enhanced reactivity in the presence of the triol clearly demonstrates its activating role in this transformation. At this point, since solvents seemed more prone to hinder than help the reaction, we conducted an experiment without any solvent (Table 1, entry 10). Gratifyingly, a 54% conversion was obtained in the presence of **2**, while no conversion at all could be observed without the activating agent. It is important to mention for this entry that even if morpholine is the only liquid component at room temperature, a homogeneous solution is generated around 60 °C by the fusion of 1,1,1-tris(hydroxymethyl)propane (melting point 56–58 °C).

**Table 1:** Initial screening.

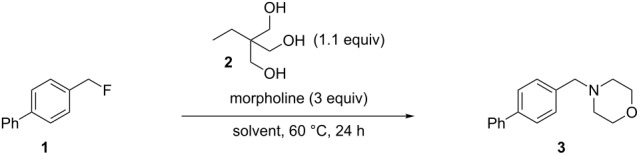			

entry	solvent	conversion (%)^a^	
	
		with **2**	without **2**

1	H_2_O	35	26
2	EtOH	29	26
3	iPrOH	16	12
4	toluene	< 3	< 3
5	AcOEt	< 3	< 3
6	THF	< 3	< 3
7	DMF	< 3	< 3
8	hexane	21	< 3
9	CH_2_Cl_2_	26	< 3
10	–	54	< 3

^a^Determined by ^1^H NMR analysis of the crude reaction mixture.

Starting from our solvent-free conditions, which were providing the better results, we envisioned that further optimization was possible to improve the yield (Table 2). First, temperature had an important impact on conversion. Indeed, going from 60 °C (Table 2, entry 1) to 100 °C (Table 2, entry 3) smoothly effected a full conversion. It was also possible to reduce the amount of morpholine used from 3 equiv (Table 2, entry 3) to 2 equiv (Table 2, entry 5) without any impact on the conversion. However, lower amounts (Table 2, entries 6 and 7) resulted in decreased conversions. While a reasonable explanation for the requirement of excess amine would be its role in capturing the HF released during the reaction, control experiments did not support this proposal. For instance, running the reaction with 1 equiv of morpholine in the presence of 1 equiv of Et_3_N provided the same conversion (Table 2, entry 8) while using inorganic bases (1 equiv of either K_3_PO_4_ or K_2_CO_3_) gave lower conversions (Table 2, entries 9 and 10). The reasons for such observations are unclear at present. Nonetheless, conditions presented in entry 5 were chosen for evaluation of the scope.

**Table 2:** Fine-tuning.

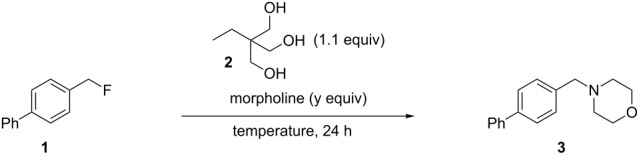			

entry	temperature	morpholine (equiv)	conversion (%)^a^

1	60	3	54
2	80	3	90
3	100	3	> 97
4	100	2.5	> 97
5	100	2	> 97 (86)^b^
6	100	1.5	91
7	100	1	70
8^c^	100	1	71
9^d^	100	1	57
10^e^	100	1	41

^a^Determined by ^1^H NMR analysis of the crude reaction mixture. ^b^Isolated yield. ^c^Et_3_N (1 equiv) was also added. ^d^K_3_PO_4_ (1 equiv) was also added. ^e^K_2_CO_3_ (1 equiv) was also added.

As shown in Table 3, reactivity is not limited to benzylic fluoride **1** and morpholine. A range of secondary amines can be used, as cyclic (Table 3, entries 1, 2, 5, 6, 8 and 11), acylic (Table 3, entries 3, 4, 9 and 10) and aromatic (Table 3, entry 7) N-nucleophiles all provide good isolated yields. In the case of *N*-methylaminoethanol, complete selectivity was observed for *N*-benzylation (Table 3, entries 4 and 10). The reaction also tolerates simple electronic variations on the benzylic fluoride, as the 4-phenyl group can be exchanged for a 4-bromo (Table 3, entries 5–7), 4-*tert*-butyl (Table 3, entries 8–10) or 3-methoxy group (Table 3, entry 11) with only minor impact on the conversion. Overall, conversion into the desired product was superior to 90% for all entries, and only the purification step proved detrimental to the isolated yields. This is further demonstrated by the reaction run at a greater scale which facilitated the purification and resulted in a higher isolated yield (Table 3, entry 1). Reactions of secondary benzylic fluorides were unfortunately untractable, and primary aliphatic amines (i.e. *n*-butylamine) generated a mixture of inseparable mono- and dibenzylation products (see Supporting Information File 1 for details).

**Table 3:** Substrate scope.

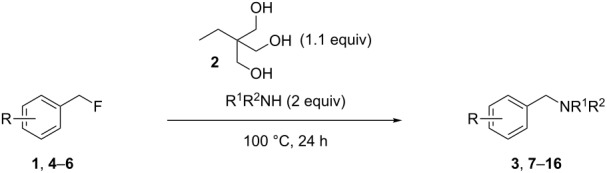				

entry	benzyl fluoride	R^1^R^2^NH	product	yield (%)^a^

1	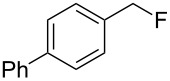 **1**	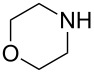	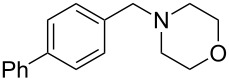 **3**	86 (97)^b^
2	**1**	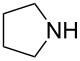	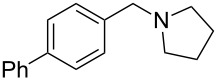 **7**	79
3	**1**	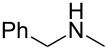	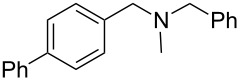 **8**	83
4	**1**	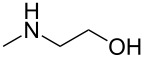	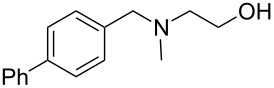 **9**	77^c^
5	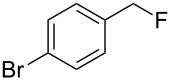 **4**	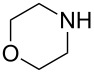	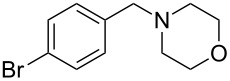 **10**	72
6	**4**	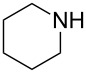	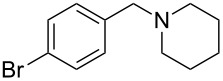 **11**	64
7	**4**	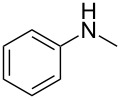	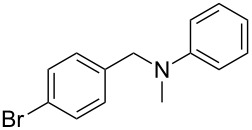 **12**	56
8	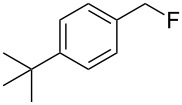 **5**	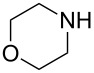	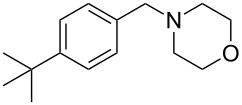 **13**	86
9	**5**	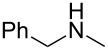	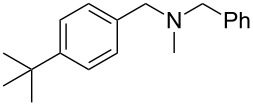 **14**	76
10	**5**	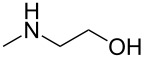	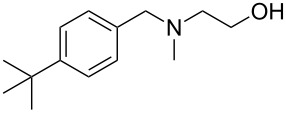 **15**	86^c^
11	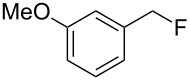 **6**	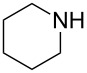	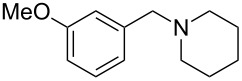 **16**	63

^a^Isolated yield for reaction on 0.16 mmol scale. ^b^Isolated yield for reaction on 0.81 mmol scale. ^c^No product of *O*-benzylation could be detected by ^1^H NMR on the crude reaction mixture.

## Conclusion

In summary, we have described that according to our previous proposed mechanism for the hydrogen bond-promoted C–F bond activation [17], simultaneous coordination of the three lone pairs of fluorine by a triol (e.g. **2**) permits the nucleophilic substitution of benzylic fluorides by amines under neutral and solvent-free conditions. To further support this hypothesis, solvent properties concerning their hydrogen bond acidity or basicity correlate well with the experimental evidence of reactivity. Investigations concerning the reaction mechanism and precise role of the three hydroxy groups of **2** for the reaction are currently underway in our laboratory.

## Supporting Information

File 1 General methods, synthetic procedures, ^1^H NMR spectra for known compounds and full characterization of all new compounds.
