# Supplementary material for: Triol-promoted activation of C–F bonds: Amination of benzylic fluorides under highly concentrated conditions mediated by 1,1,1-tris(hydroxymethyl)propane
Source: Beilstein J Org Chem. 2013 Nov 13;9:2451–6. doi: 10.3762/bjoc.9.283 (PMC3869259; doi:10.3762/bjoc.9.283)

Supporting Information

for

Triol-promoted activation of C–F bonds: Amination of benzylic fluorides under highly concentrated conditions mediated by 1,1,1-tris(hydroxymethyl)propane

Pier Alexandre Champagne, Alexandre Saint-Martin, Mélina Drouin and Jean-François Paquin*

Address: Canada Research Chair in Organic and Medicinal Chemistry, CCVC, PROTEO, Département de chimie, Université Laval, 1045 avenue de la Médecine, Québec, QC, Canada G1V 0A6

Email: Jean-Francois Paquin - jean-francois.paquin@chm.ulaval.ca

*Corresponding author

General methods, synthetic procedures, ^{1}H NMR spectra for known compounds and full characterization of all new compounds

Table of contents

General information .... Page S2

Materials and methods .... Page S3

Synthesis of fluorinated substrates .... Page S3

Substitution reactions .... Page S6

Other attempted reactions .... Page S12

^{1}H NMR spectra of known compounds .... Page S13

NMR spectra of all new compounds .... Page S26

General information

Unless otherwise noted, all commercial reagents were used without further purification. Dichloromethane, toluene, ether, tetrahydrofuran and acetonitrile were purified using a Vacuum Atmospheres Inc. Solvant Purification System. Thin-layer chromatography (TLC) analysis of reaction mixtures was performed using Silicyle silica gel 60 Å F254 TLC plates, and visualized under UV or by staining with iodine. Flash column chromatography was carried out on Silicycle Silica Gel 60 Å, 230 × 400 mesh. High-resolution mass spectra were obtained on a LC/MS–TOF Agilent 6210 using either electrospray ionization (ESI) or atmospheric pressure photoionization (APPI). Nuclear magnetic resonance (NMR) spectra were recorded using Agilent DD2 500 and Varian Inova 400 spectrometers. ^{1}H and ^{13}C chemical shifts are reported in ppm downfield of tetramethylsilane and referenced to tetramethylsilane (δ = 0 ppm) or residual chloroform peak (δ = 7.26 ppm). For ^{19}F NMR, $CFCl_3$ is used as the external standard. Coupling constants (*J*) are measured in hertz (Hz). Multiplicities are reported using the following abbreviations: s = singlet, d = doublet, t = triplet, q = quartet, m = multiplet, br = broad resonance. Infrared spectra were recorded using a Thermo Scientific Nicolet 380 FTIR spectrometer. Melting points were recorded on a Stanford ResearchSystem OptiMelt capillary melting point apparatus and are uncorrected.

Materials and methods

Synthesis of fluorinated substrates

4-(Fluoromethyl)-1,1'-biphenyl (**1**), 1-bromo-4-(fluoromethyl)benzene (**4**) and 1-*tert*-butyl-4-(fluoromethyl)benzene (**5**) were synthesised according to a literature protocol.[1]

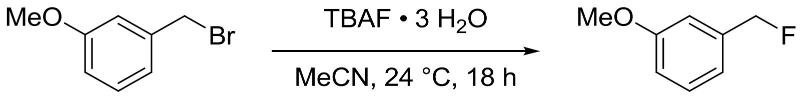

6

1-(Fluoromethyl)-3-methoxybenzene (6). In a round-bottomed flask, commercially available 3-methoxybenzyl bromide (750 mg, 3.73 mmol, 1 equiv) was diluted in MeCN (15 mL, c = 0.25 M). The flask was purged with argon, then tetrabutylammonium fluoride trihydrate (2.06 g, 6.53 mmol, 1.75 equiv) was added and the reaction mixture was allowed to stir for 18 hours at room temperature. The reaction was quenched with 50 mL of water and the solution was extracted with 3 × Et_2O. Combined organic extracts were washed with brine, dried over anhydrous $MgSO_4$, filtered and evaporated under reduced pressure. After silica gel chromatography using hexanes/EtOAc (95:5), product-containing fractions were filtered over cotton wool prior to evaporation into a polypropylene tube. The title compound (422 mg, 81%) was obtained as a clear liquid.

^{1}H NMR (400 MHz, $CDCl_3$) δ 3.82 (s, 3H), 5.36 (d, J = 47.7 Hz, 2H), 6.89-6.96 (m, 3H), 7.30 (t, J = 7.8 Hz, 1H); ^{19}F NMR (376 MHz, $CDCl_3$) δ -207.9 (t, J = 47.7 Hz); ^{13}C NMR (126 MHz, $CDCl_3$) δ 55.4, 84.6 (d, J = 167.3 Hz), 112.8 (d, J = 6.3 Hz), 114.5 (d, J = 2.9

[1] Champagne, P. A.; Pomarole, J.; Thérien, M.-È.; Benhassine, Y.; Beaulieu, S.; Legault, C. Y.; Paquin, J.-F. *Org. Lett.* **2013**, *15*, 2210.

Hz), 119.6 (d, J = 6.2 Hz), 129.8 (d, J = 0.8 Hz), 137.9 (d, J = 17.1 Hz), 159.9; IR (ATR, ZnSe) ν = 2958, 1588, 1490, 1457, 1267, 1156, 1041, 978, 781, 738, 692 cm $^{-1}$; HRMS-APPI calcd for C_8H_9FO $[M^*]^+$ 140.0632, found 140.0645.

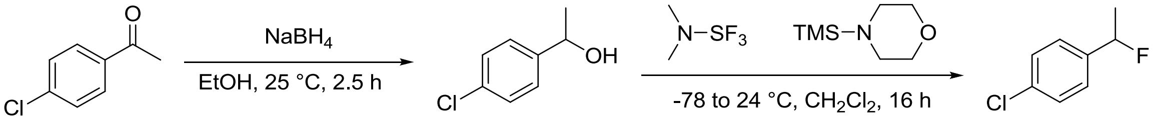

1-(4-Chlorophenyl)ethanol (SM-1). In a round-bottomed flask, commercially available 4-chloroacetophenone (1.00 g, 6.47 mmol, 1 equiv) was diluted in absolute ethanol (3 mL), then a suspension of sodium borohydride (162 mg, 4.27 mmol, 0.66 equiv) in 7 mL of absolute EtOH was added. The reaction mixture was allowed to stir for 2.5 hours, upon which the reaction appeared completed by TLC analysis. The reaction mixture was quenched with aq. 10% NaOH and stirred until the solution was homogeneous. Water was added and ethanol was evaporated under reduced pressure. The aqueous mixture was extracted with 3 × CH_2Cl_2 and the combined organic extracts were washed with $NaHCO_3$ (aq. 5%), then water. The solution was dried over anhydrous Na_2SO_4, filtered and concentrated in vacuo, affording the pure title compound (1.04 g, 100%) as a clear liquid. Spectral data were identical to those previously reported.[2]

1-Chloro-4-(1-fluoroethyl)benzene (SM-2). This compound was synthesised by a variation of a known protocol.[3,4] In a 50 mL round-bottomed flask, a solution of (dimethylamino)sulfur trifluoride (Me-DAST, 523 µL, 4.79 mmol, 1.5 equiv) in 5 mL

[2] Wei, Y.; Xue, D.; Lei, Q.; Wang, C.; Xiao, J. *Green Chem.* **2013**, *15*, 629.
[3] Bio, M. M.; Waters, M.; Javadi, G.; Song, Z. J.; Zhang, F.; Thomas, D. *Synthesis* **2008**, 891.
[4] Bresciani, S.; O'Hagan, D. *Tetrahedron Lett.* **2010**, *51*, 5795.

CH_2Cl_2 was cooled to −78 °C, upon which 4-(trimethylsilyl)morpholine (935 μL, 5.26 mmol, 1.65 equiv) was added. The mixture was allowed to heat to room temperature and stirred for 2.5 hours. This solution was cooled again to −78 °C and a solution of 1-(4-chlorophenyl)ethanol **(SM-1)** (500 mg, 3.19 mmol, 1 equiv) in 10 mL CH_2Cl_2 was added dropwise. The mixture was again allowed to reach room temperature and was stirred for 16 hours, at which point it was quenched by slowly adding 1 mL MeOH, then a saturated aqueous $NaHCO_3$ solution. The aqueous mixture was extracted with 3 × CH_2Cl_2 and the combined organic extracts were dried over anhydrous $MgSO_4$, filtered and concentrated. After silica-gel chromatography using 100% hexanes, product-containing fractions were filtered over cotton wool prior to evaporation into a polypropylene tube. The title compound (274 mg, 54%) was obtained as a colorless oil. Spectral data were identical to those previously reported.[5]

[5] Liu, W.; Groves, J. T. *Angew. Chem. Int. Ed.* **2013**, *52*, 6024.

Substitution reactions

General procedure:

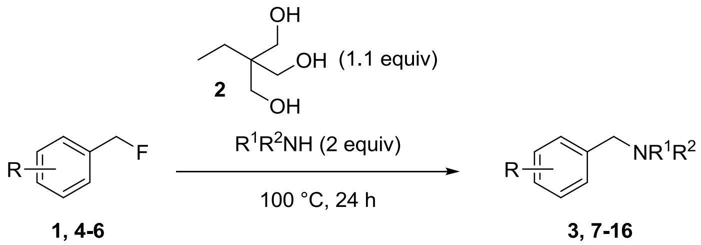

All amination reactions of the article were run using the following procedure. In the initial screening and fine-tuning sections, solvent (0.5 M concentration) was used and reaction conditions were modified from the described procedure below.

In a Biotage microwave vial (which serves as a general borosilicate glass vessel) equipped with a magnetic stir bar, the benzylic fluoride (1 equiv) was weighted and then freshly ground 1,1,1-tris(hydroxymethyl)propane (**2**) (1.1 equiv) was added. The amine (2 equiv) was added through a syringe and finally, the vial was capped and purged with argon. The reaction was placed in an oil bath and stirred at 100 °C for 24 hours. At the end of the heating period, an aqueous Na_2CO_3 (1 M) solution was added (on the typical scale, 1 mL was used) and the reaction mixture was stirred to loosen up its pasty form. This mixture was then extracted with 3 × Et_2O and the combined organic extracts were washed with brine, dried on anhydrous $MgSO_4$, filtered and evaporated in vacuo. At this point, conversion was measured by ^1H NMR. Silica gel column chromatography followed when necessary.

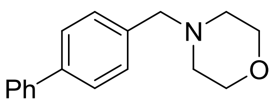

4-(Biphenyl-4-ylmethyl)morpholine (3). Following the general procedure on a 0.16 mmol (30 mg) scale of 4-(fluoromethyl)-1,1'-biphenyl (**1**) and using freshly distilled morpholine (28 μL, 0.32 mmol), 99% of conversion is measured by ^{1}H NMR after work-up. Upon column chromatography (70:30 hexanes/AcOEt), the product (35 mg, 86%) was obtained as a yellowish oil. Spectral data were identical to those previously reported.[6]

The same reaction was run using a 150 mg (0.81 mmol) scale of **1**. For this experiment, the product (199 mg, 97%) was obtained in the pure form using the same purification conditions described above.

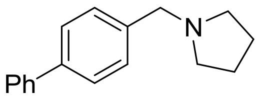

1-(Biphenyl-4-ylmethyl)pyrrolidine (7). Following the general procedure on a 0.16 mmol (30 mg) scale of 4-(fluoromethyl)-1,1'-biphenyl (**1**) and using freshly distilled pyrrolidine (27 μL, 0.32 mmol), 91% of conversion is measured by ^{1}H NMR after work-up. The product (30 mg, 79%) was isolated as a brown oil using silica gel chromatography (90:10 CH_2Cl_2/MeOH with 0.1% Et_3N). Spectral data were identical to those previously reported.[1]

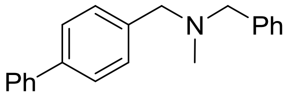

***N*-Benzyl-1-(biphenyl-4-yl)-*N*-methylmethanamine (8).** Following the general procedure on a 0.16 mmol (30 mg) scale of 4-(fluoromethyl)-1,1'-biphenyl (**1**) and using freshly distilled *N*-benzylmethylamine (41 μL, 0.32 mmol), 92% of conversion is measured by ^{1}H NMR after work-up. Upon

[6] Blessley, G.; Holden, P.; Walker, M.; Brown, J. M.; Gouverneur, V. *Org. Lett.* **2012**, *14*, 2754.

column chromatography (90:10 hexanes/AcOEt), the product (38 mg, 83%) was obtained as a colorless oil. Spectral data were identical to those previously reported.[1]

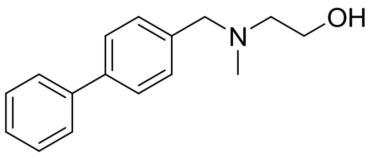

2-((Biphenyl-4-ylmethyl)(methyl)amino)ethanol (9). Following the general procedure on a 0.16 mmol (30 mg) scale of 4-(fluoromethyl)-1,1'-biphenyl (**1**) and using freshly distilled 2-(methylamino)ethanol (26 µL, 0.32 mmol), 95% of conversion is measured by ^{1}H NMR after work-up. The product (30 mg, 77%) is obtained as a colorless oil by flash chromatography (80/20 CH_2Cl_2/MeOH with 0.1% Et_3N). ^{1}H NMR ($CDCl_3$, 400 MHz) δ 2.35 (s, 3H), 2.71 (t, *J* = 5.1 Hz, 2H), 3.71 (s, 4H), 3.87 (br s, 1H), 7.34-7.47 (m, 5H), 7.57-7.61 (m, 4H); ^{13}C NMR ($CDCl_3$, 126 MHz) δ 41.6, 58.40, 58.42, 62.0, 127.2, 127.3, 127.4, 128.9, 129.7, 137.1, 140.4, 140.9; IR (ATR, ZnSe) ν = 3409, 2924, 1361, 1220, 1031, 759, 697 cm^{-1}; HRMS-ESI calcd for $C_{16}H_{19}NO$ $[M+H]^+$ 242.1539, found 242.1538.

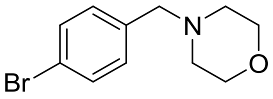

4-(4-Bromobenzyl)morpholine (10). Following the general procedure on a 0.152 mmol (28.5 mg) scale of 1-bromo-4-(fluoromethyl)benzene (**4**) and using freshly distilled morpholine (28 µL, 0.32 mmol), 100% conversion is measured by ^{1}H NMR after work-up. Upon silica gel chromatography, the product (28 mg, 72%) was isolated as a white solid by column

chromatography using hexanes/EtOAc (70:30). Spectral data were identical to those previously reported.[7]

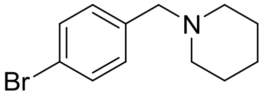

1-(4-Bromobenzyl)piperidine (11). Following the general procedure on a 0.16 mmol (30 mg) scale of 1-bromo-4-(fluoromethyl)benzene (**4**) and using freshly distilled piperidine (32 μL, 0.32 mmol), 100% conversion is measured by ^{1}H NMR after work-up. The product (26 mg, 64%) was obtained as a yellowish oil by flash chromatography using CH_2Cl_2/MeOH (90:10). Spectral data were identical to those previously reported.[8]

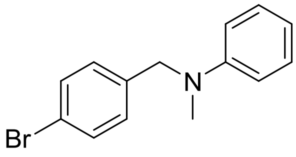

***N*-(4-Bromobenzyl)-*N*-methylaniline (12).** *Prior to use, N-methylaniline was purified by extraction. Solubilised in Et_2O, it was extracted with 3 × HCl (10%) and the combined aqueous phases were lowered to pH 12 using aqueous (1 M) Na_2CO_3. This aqueous phase was extracted with 3 × Et_2O, and the combined organic extracts were dried over $MgSO_4$, filtered and evaporated to yield pure N-methylaniline.* Following the general procedure on a 0.16 mmol (30 mg) scale of 1-bromo-4-(fluoromethyl)benzene (**4**) and using purified (see above) *N*-methylaniline (35 μL, 0.32 mmol), 90% conversion is measured by ^{1}H NMR after work-up. After silica gel chromatography using hexanes/EtOAc (96/4),

[7] Kumar, V.; Sharma, U.; Verma, P. K.; Kumar, N.; Singh, B. *Adv. Synth. Catal.* **2012**, *354*, 870.
[8] Das, S.; Addis, D.; Zhou, S.; Junge, K.; Beller, M. *J. Am. Chem. Soc.* **2010**, *132*, 1770.

the compound (25 mg, 56%) was obtained as a colorless oil. Spectral data were identical to those previously reported.[9]

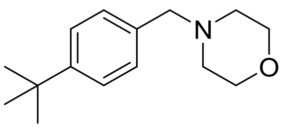

4-(4-*tert*-Butylbenzyl)morpholine (13). Following the general procedure on a 0.165 mmol (28 mg) scale of 1-*tert*-butyl-4-(fluoromethyl)benzene (**5**) and using freshly distilled morpholine (28 μL, 0.32 mmol), 100% conversion is measured by ^{1}H NMR after work-up. Product (33 mg, 86%) was obtained as a yellowish oil, pure after work-up. Spectral data were identical to those previously reported.[10]

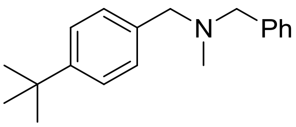

***N*-Benzyl-1-(4-*tert*-butylphenyl)-*N*-methylmethanamine (14).** Following the general procedure on a 0.16 mmol (27 mg) scale of 1-*tert*-butyl-4-(fluoromethyl)benzene (**5**) and using freshly distilled *N*-benzylmethylamine (41 μL, 0.32 mmol), 99% conversion is measured by ^{1}H NMR after work-up. The product (33 mg, 76%) was purified by flash chromatography using hexanes/EtOAc (90:10) and is a colorless liquid. ^{1}H NMR (400 MHz, $CDCl_3$) δ 1.31 (s, 9H), 2.18 (s, 3H), 3.49 (s, 2H), 3.51 (s, 2H), 7.21-7.37 (m, 9H); ^{13}C NMR (126 MHz, $CDCl_3$) δ 31.5, 34.6, 42.4, 61.6, 61.9, 125.2, 127.0, 128.3, 128.7, 129.0, 136.3, 139.5, 149.9; IR (ATR, ZnSe) ν = 2961, 2784, 1453, 1363, 1017, 732, 697 cm^{-1}; HRMS-ESI calcd for $C_{19}H_{26}N$ [M+H]$^+$ 268.2060, found 268.2073.

---

[9] Sousa, S. C. A.; Fernandes, A. C. *Adv. Synth. Catal.* **2010**, *352*, 2218.
[10] Zhang, M.; Moore, J. D.; Flynn, D. L.; Hanson, P. R. *Org. Lett.* **2004**, *6*, 2657.

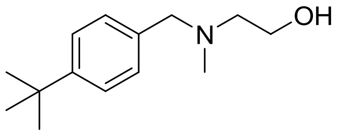

2-((4-*tert*-Butylbenzyl)(methyl)amino)ethanol (15). Following the general procedure on a 0.16 mmol (27 mg) scale of 1-*tert*-butyl-4-(fluoromethyl)benzene (**5**) and using freshly distilled 2-(methylamino)ethanol (26 μL, 0.32 mmol), 100% conversion is measured by ^{1}H NMR after work-up. The desired compound (31 mg, 86%) is obtained as a colorless oil, pure after work-up. ^{1}H NMR (400 MHz, $CDCl_3$) δ 1.31 (s, 9H), 2.23 (s, 3H), 2.59 (t, *J* = 5.4 Hz, 2H), 2.83 (br s, 1H), 3.53 (s, 2H), 3.62 (t, *J* = 5.4 Hz, 2H), 7.22 (d, *J* = 8.0 Hz, 2H), 7.34 (d, *J* = 8.0 Hz, 2H); ^{13}C NMR (126 MHz, $CDCl_3$) δ 31.5, 34.6, 41.6, 58.4, 58.5, 62.0, 125.3, 128.8, 125.5, 150.2; IR (ATR, ZnSe) ν = 3400, 2959, 1460, 1363, 1268, 1031, 1017, 810 cm^{-1}; HMRS-ESI calcd for $C_{14}H_{24}NO$ $[M+H]^+$ 222.1852, found 222.1859.

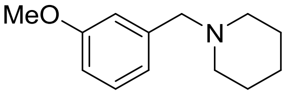

1-(3-Methoxybenzyl)piperidine (16). Following the general procedure on a 0.17 mmol (24 mg) scale of 1-(fluoromethyl)-3-methoxybenzene (**6**) and using freshly distilled piperidine (32 μL, 0.32 mmol), 98% conversion is measured by ^{1}H NMR after work-up. After silica gel chromatography using CH_2Cl_2/MeOH (90:10), the product (22 mg, 63%) was obtained as a yellowish oil. ^{1}H NMR (400 MHz, $CDCl_3$) δ 1.40-1.45 (m, 2H), 1.57-1.63 (m, 4H), 2.42 (br s, 4H), 3.49 (s, 2H), 3.81 (s, 3H), 6.79-6.81 (m, 1H), 6.90-6.92 (m 2H), 7.22 (t, *J* = 7.8 Hz, 1H); ^{13}C NMR (100 MHz, $CDCl_3$) δ 24.3, 25.8, 54.5, 55.4, 63.7, 112.7, 114.9, 121.8, 129.2, 139.7, 159.7; IR (ATR, ZnSe) ν = 2932, 1601, 1586, 1488, 1454, 1343, 1260, 1039, 772, 693 cm^{-1}; HRMS-ESI calcd for $C_{13}H_{20}NO$ $[M+H]^+$ 206.1539, found 206.1544.

Other attempted reactions

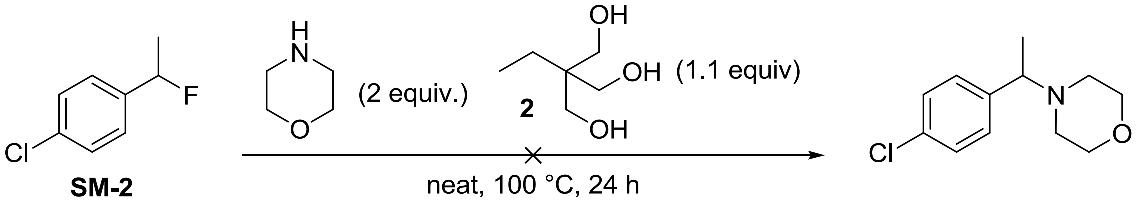

This reaction was attempted by following the general procedure for substitution reactions on a 0.16 mmol (25 mg) scale of 1-chloro-4-(1-fluoroethyl)benzene (**SM-2**) and using freshly distilled morpholine (28 μL, 0.32 mmol). However, after work-up, only 3 mg of crude was obtained and ^{1}H NMR analysis did not allow to identify any of the products formed. It is probable in these conditions that the starting fluoride (**SM-2**) decomposed and polymerized, hindering its isolation.

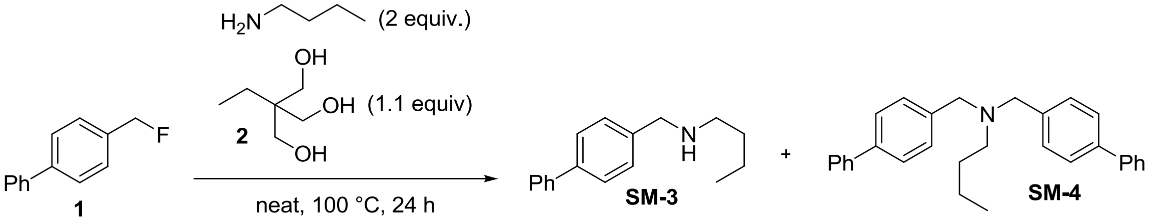

This reaction was attempted by following the general procedure for substitution reactions on a 0.16 mmol (30 mg) scale of 4-(fluoromethyl)-1,1'-biphenyl (**1**) and using freshly distilled *n*-butylamine (32 μL, 0.32 mmol). After work-up, 69% of conversion was measured by ^{1}H NMR, but two different products (**SM-3** and possibly **SM-4**) were formed in a 2:1 mixture. The purification was attempted by column chromatography using hexanes/EtOAc (96/4) but the products of mono- and dibenzylation could not be separated.

^{1}H NMR spectra of known compounds

Figure S1: **^{1}H NMR spectrum of compound 1.**

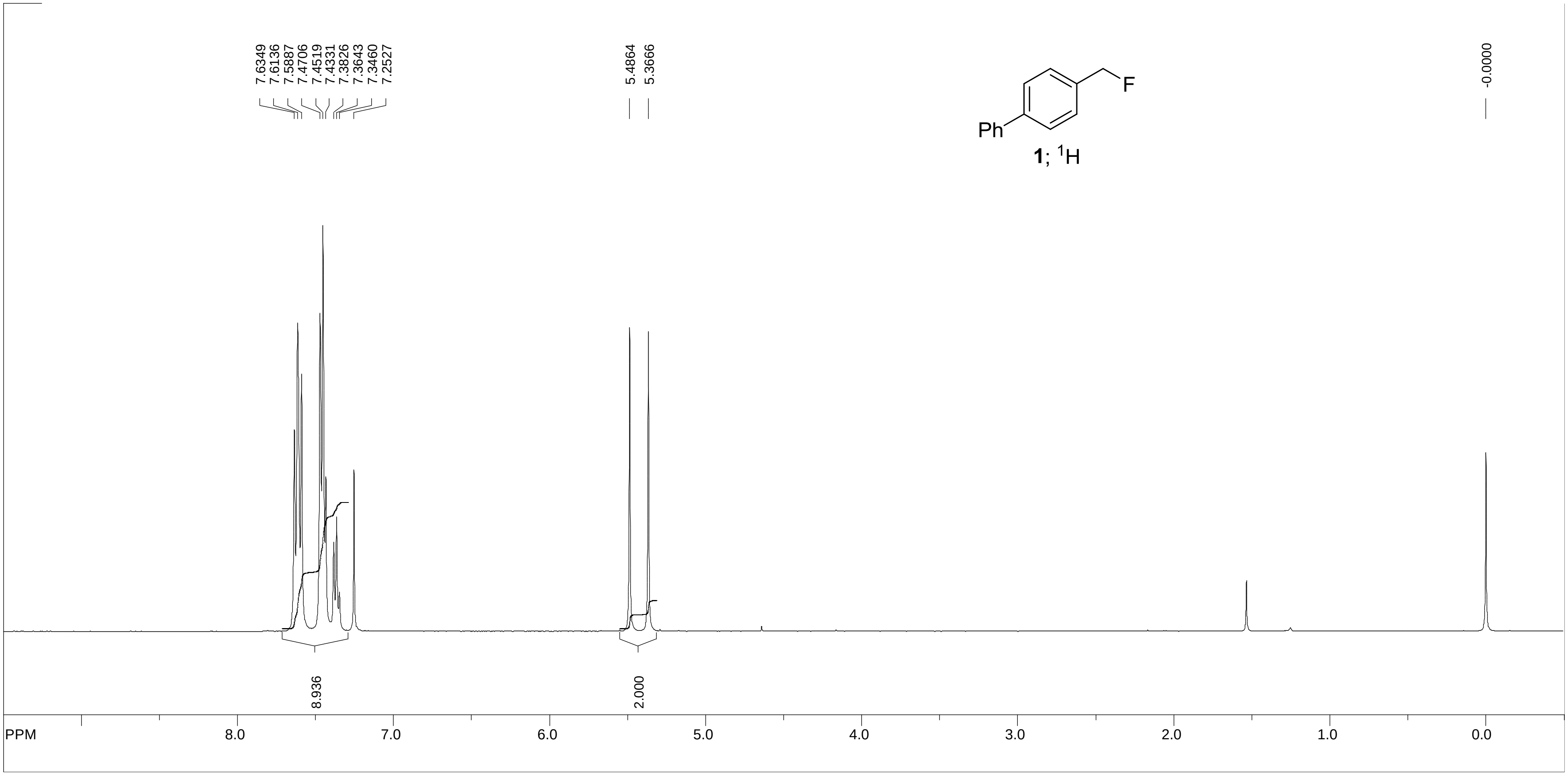

Figure S2: **^{1}H NMR spectrum of compound 3.**

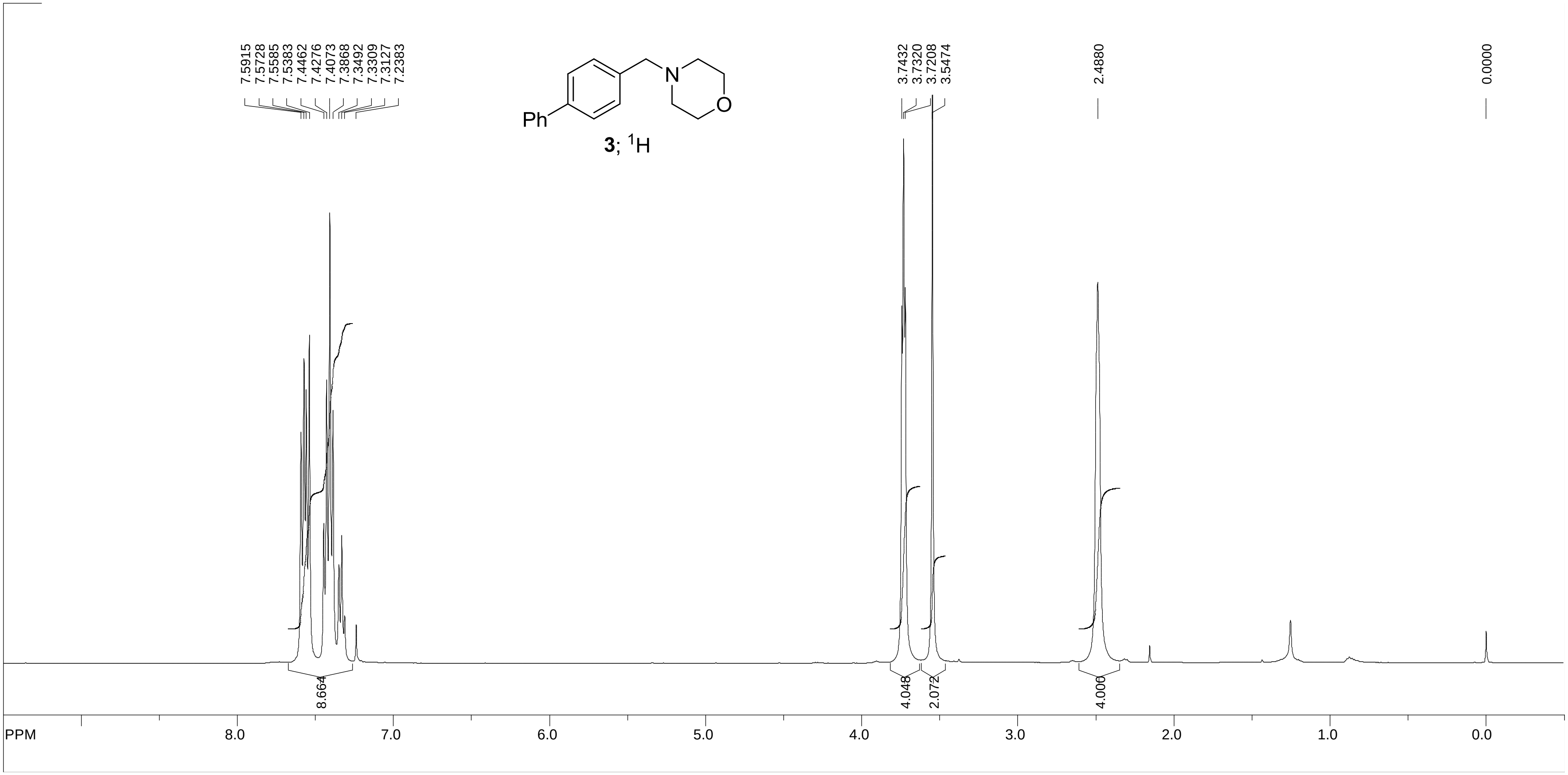

Figure S3: **^{1}H NMR spectrum of compound 4.**

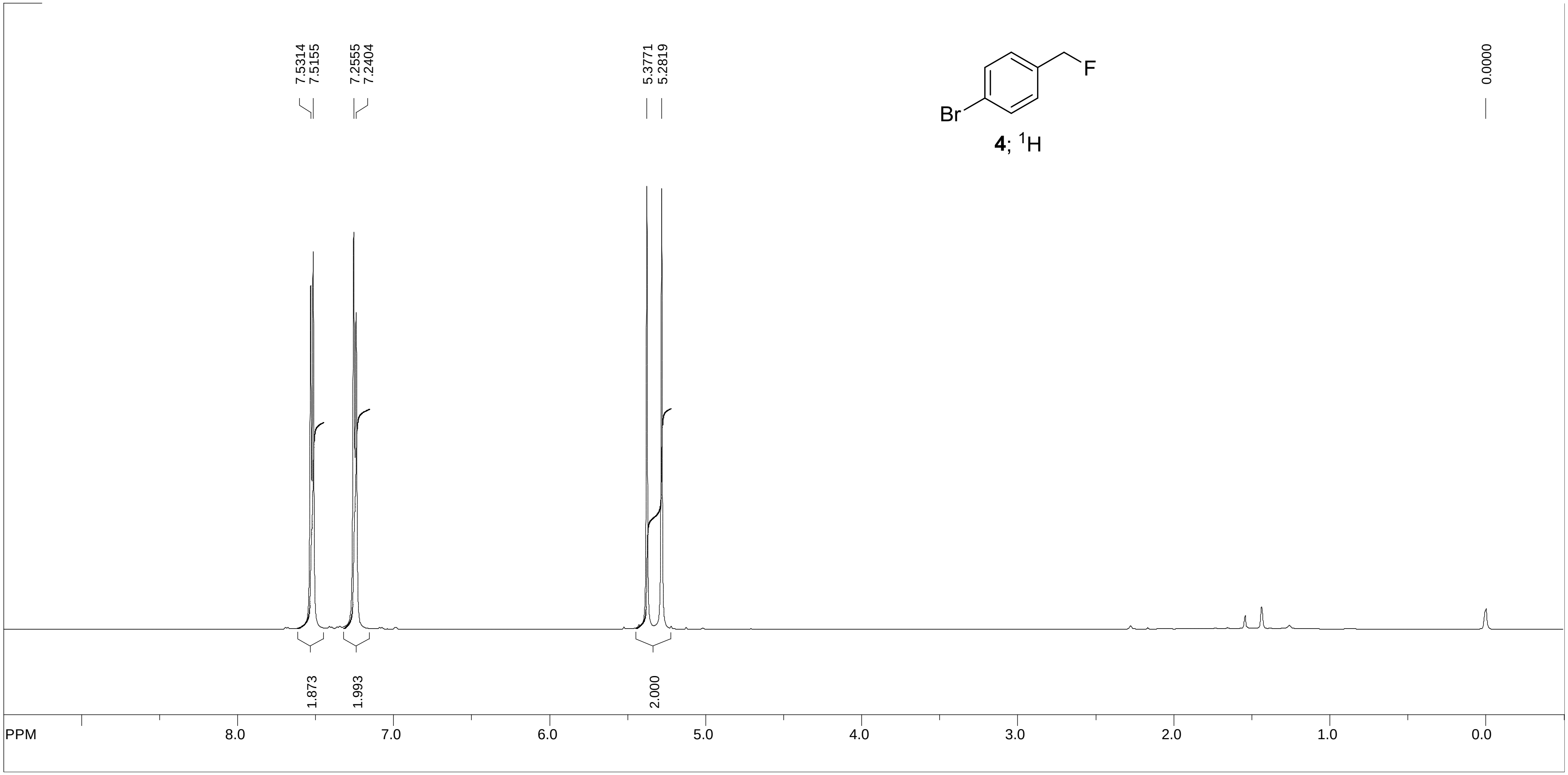

Figure S4: **^{1}H NMR spectrum of compound 5.**

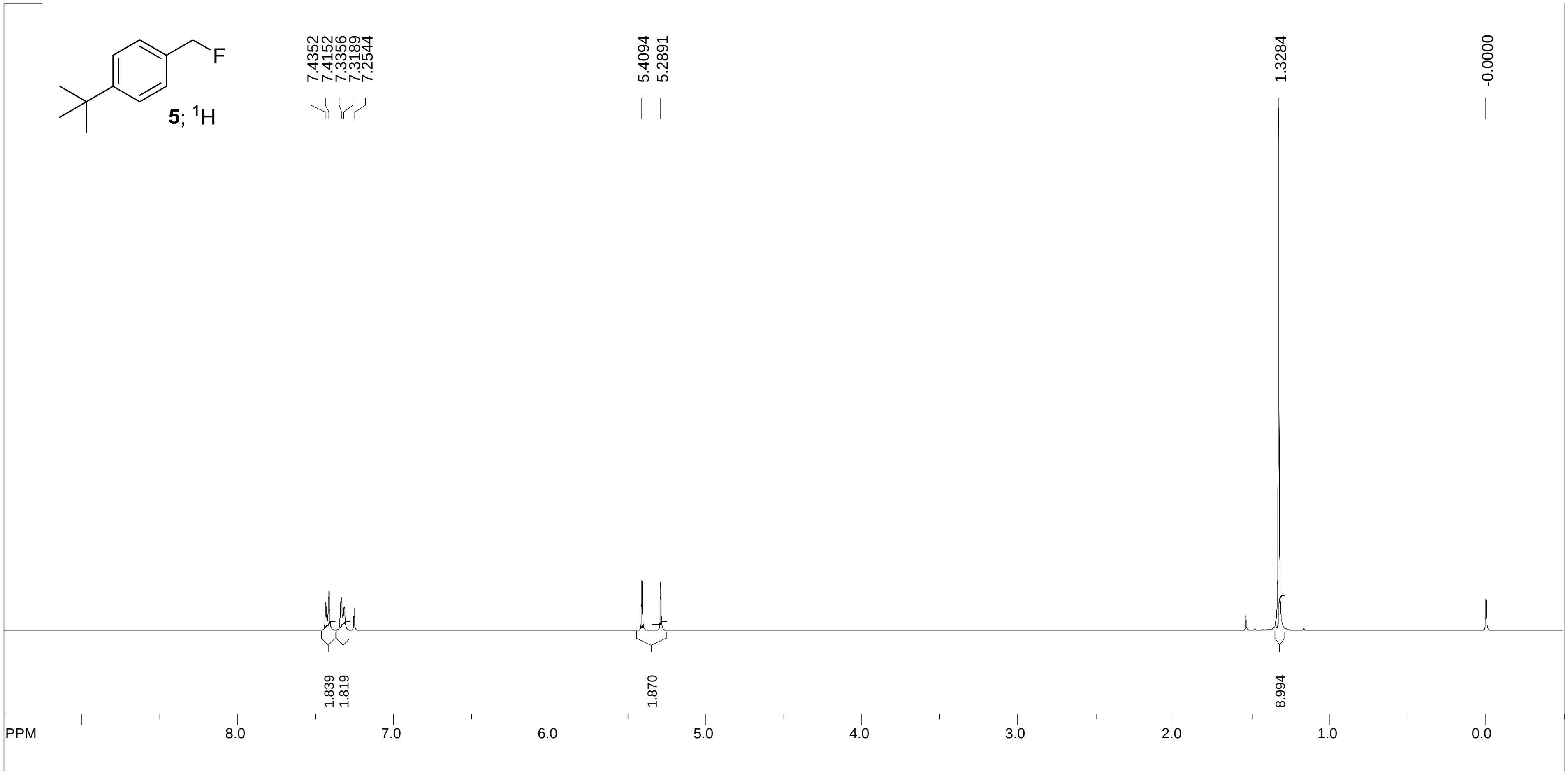

Figure S5: **^{1}H NMR spectrum of compound 7.**

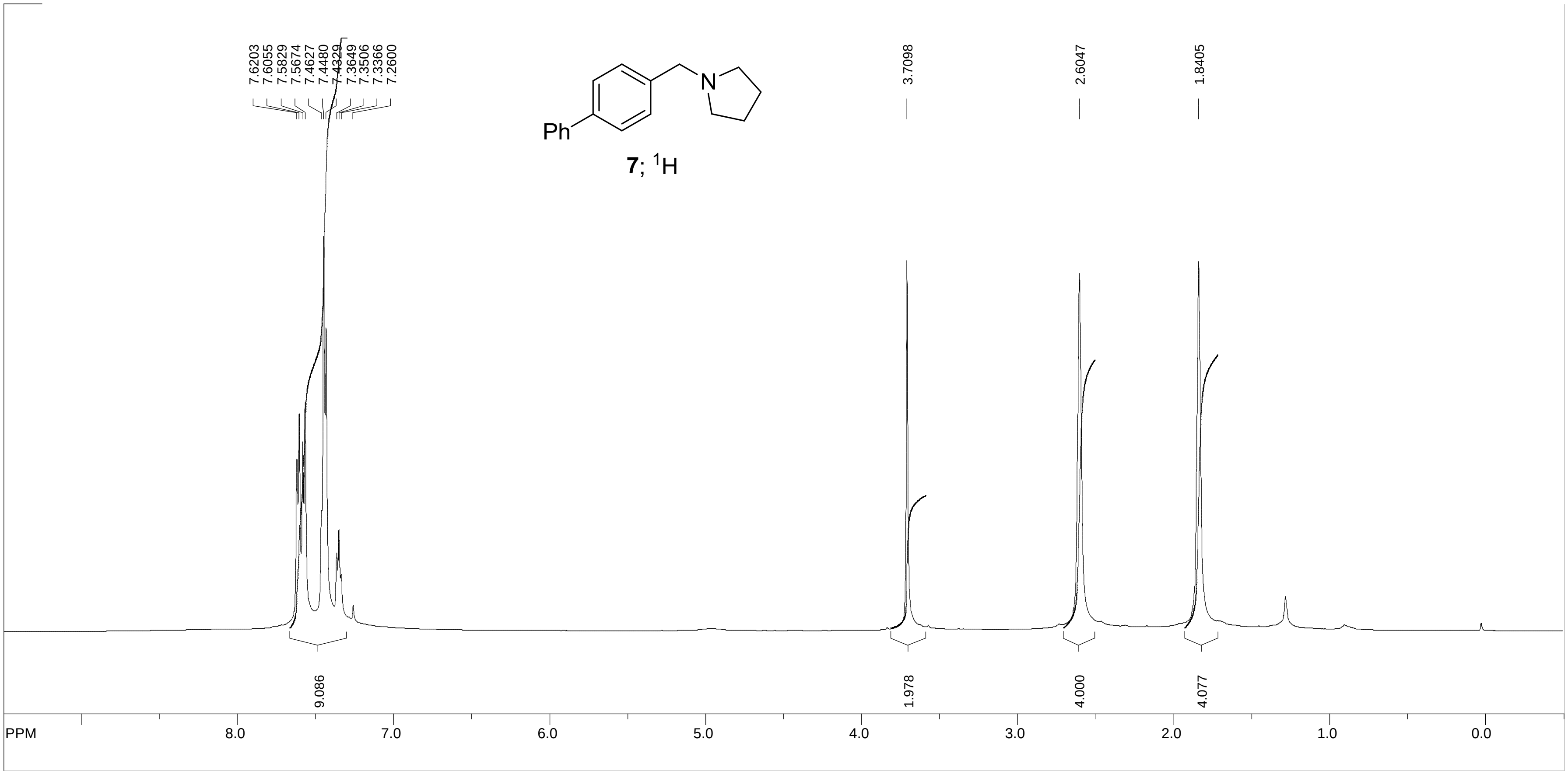

Figure S6: **^{1}H NMR spectrum of compound 8.**

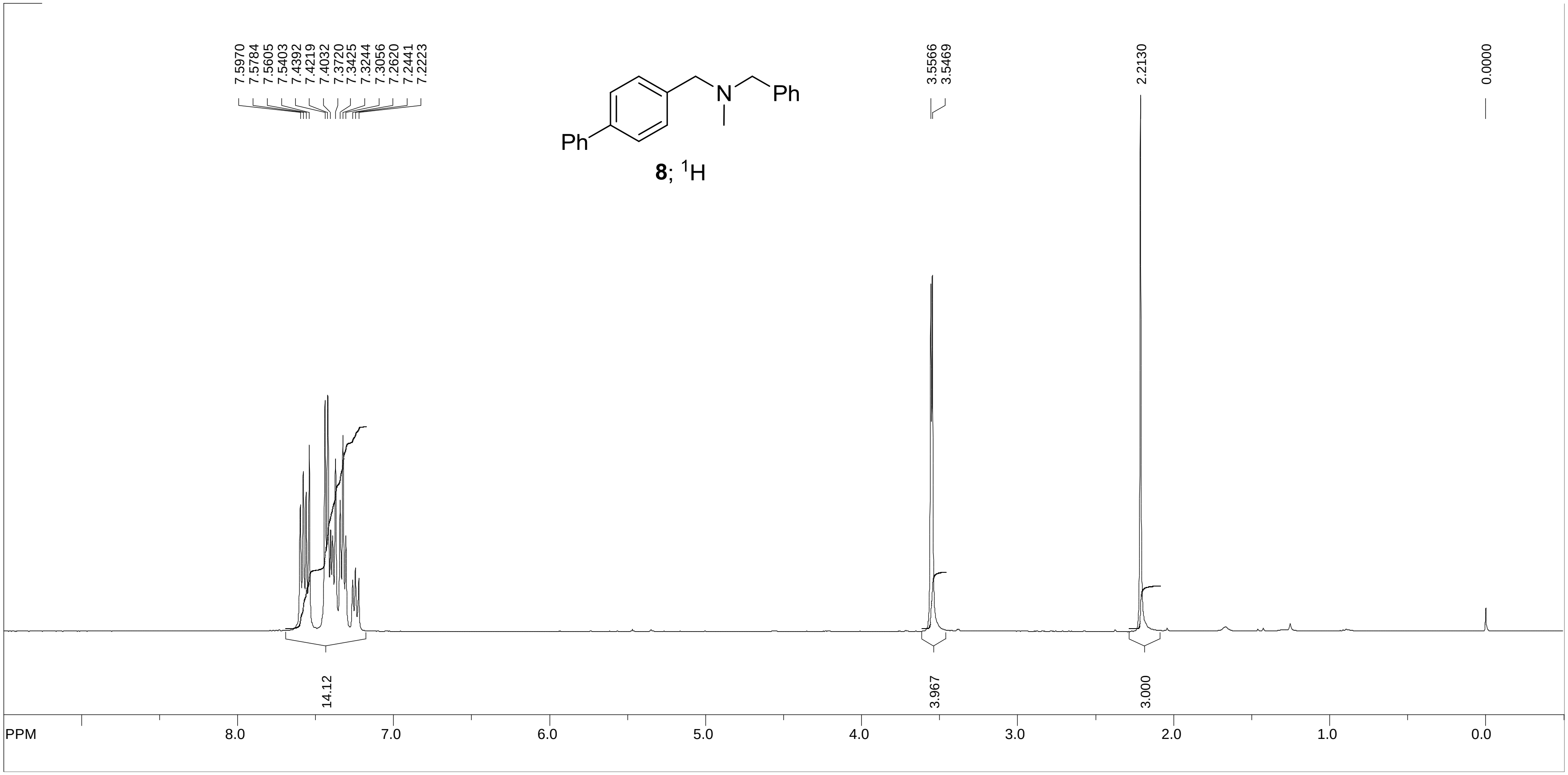

Figure S7: **^{1}H NMR spectrum of compound 10.**

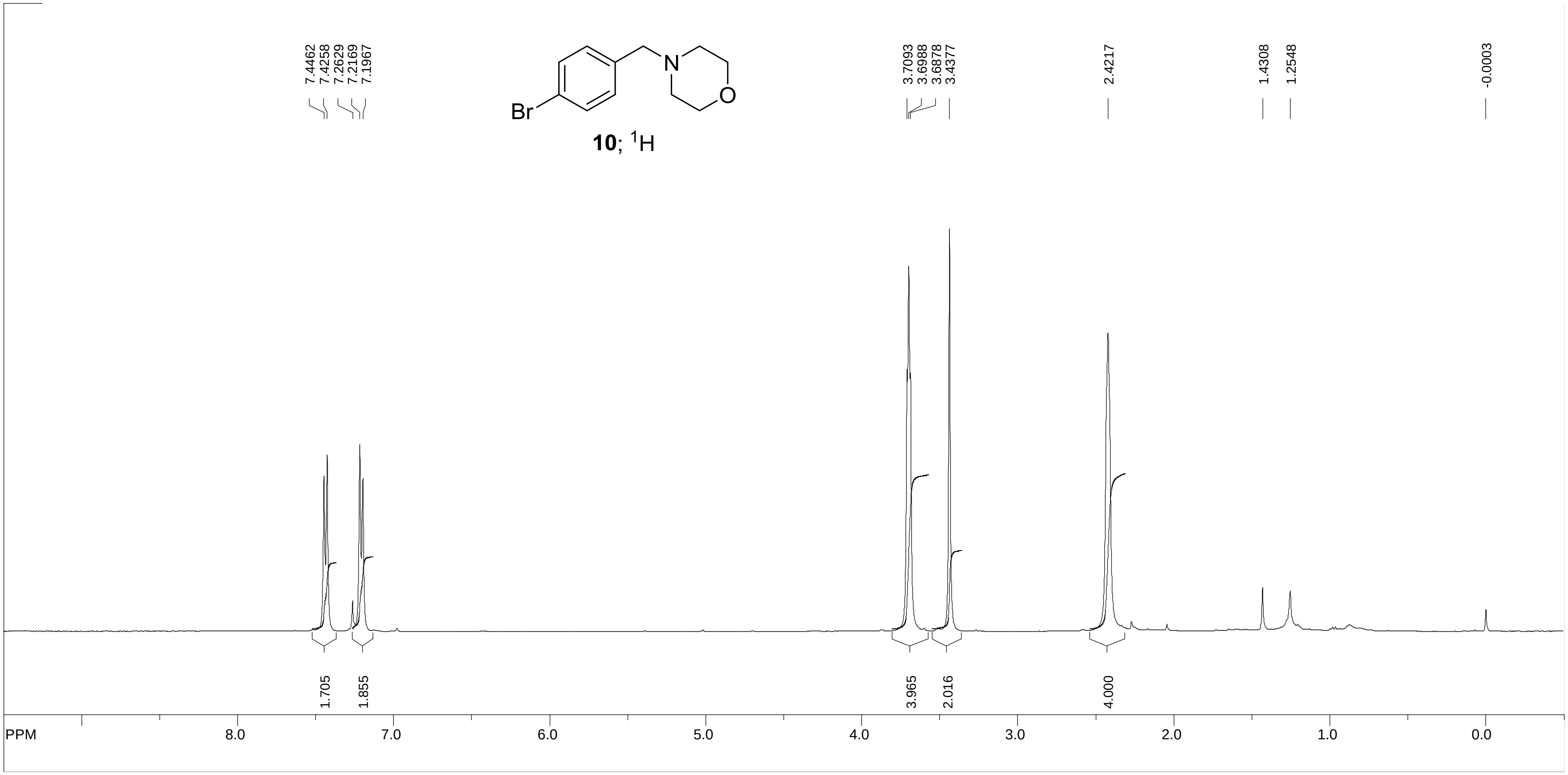

Figure S8: **^{1}H NMR spectrum of compound 11.**

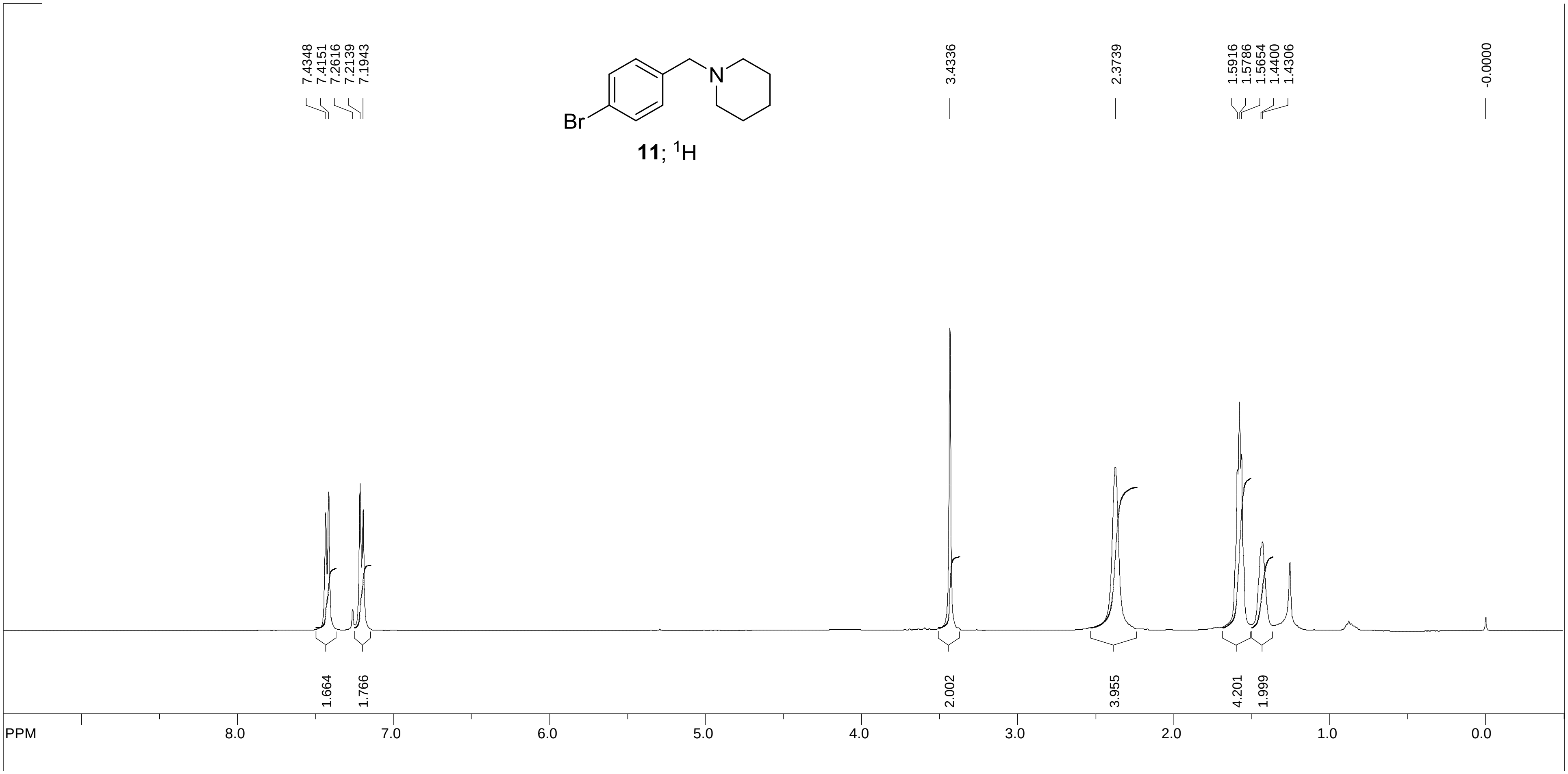

Figure S9: **^{1}H NMR spectrum of compound 12.**

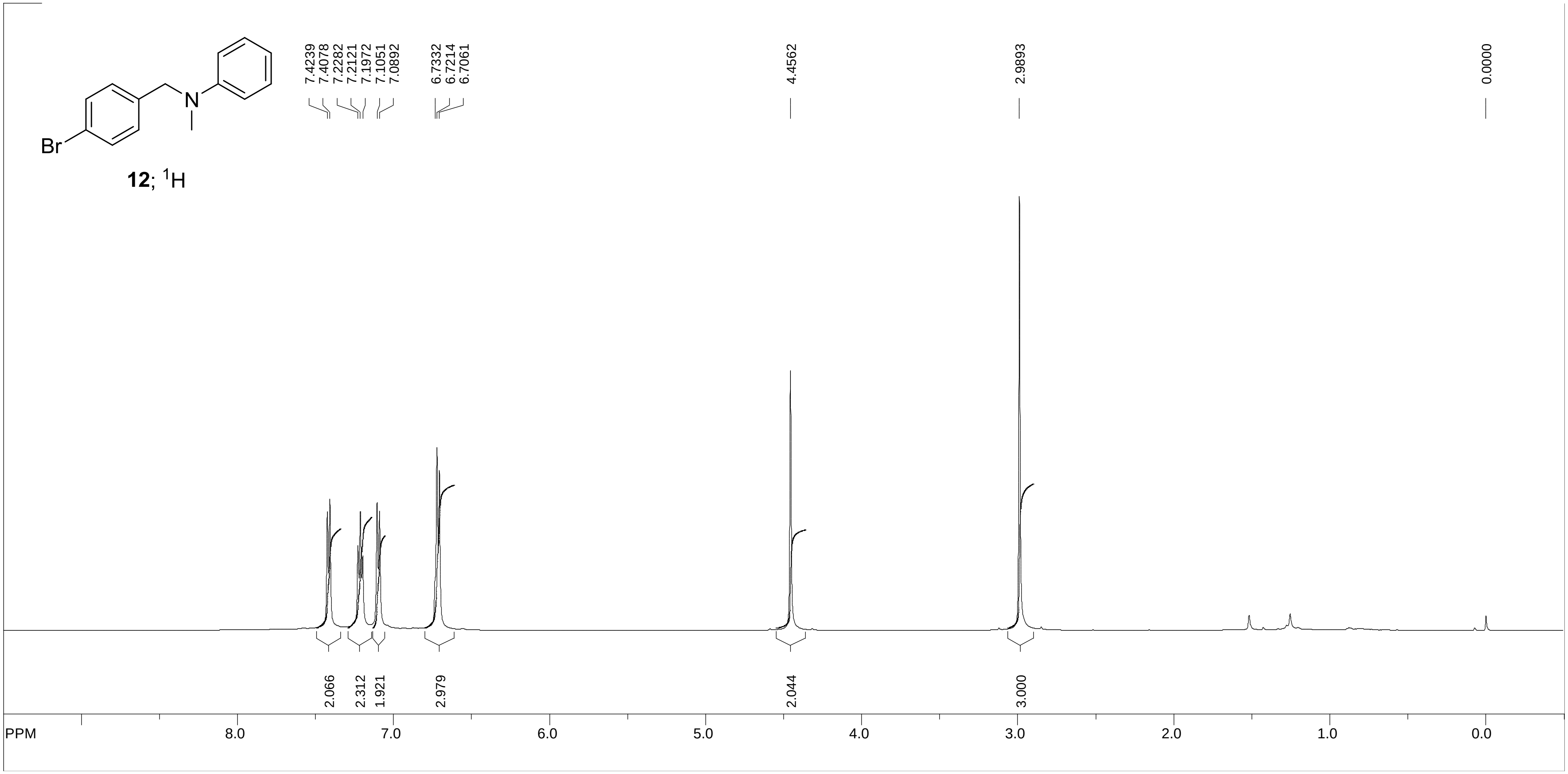

Figure S10: **^{1}H NMR spectrum of compound 13.**

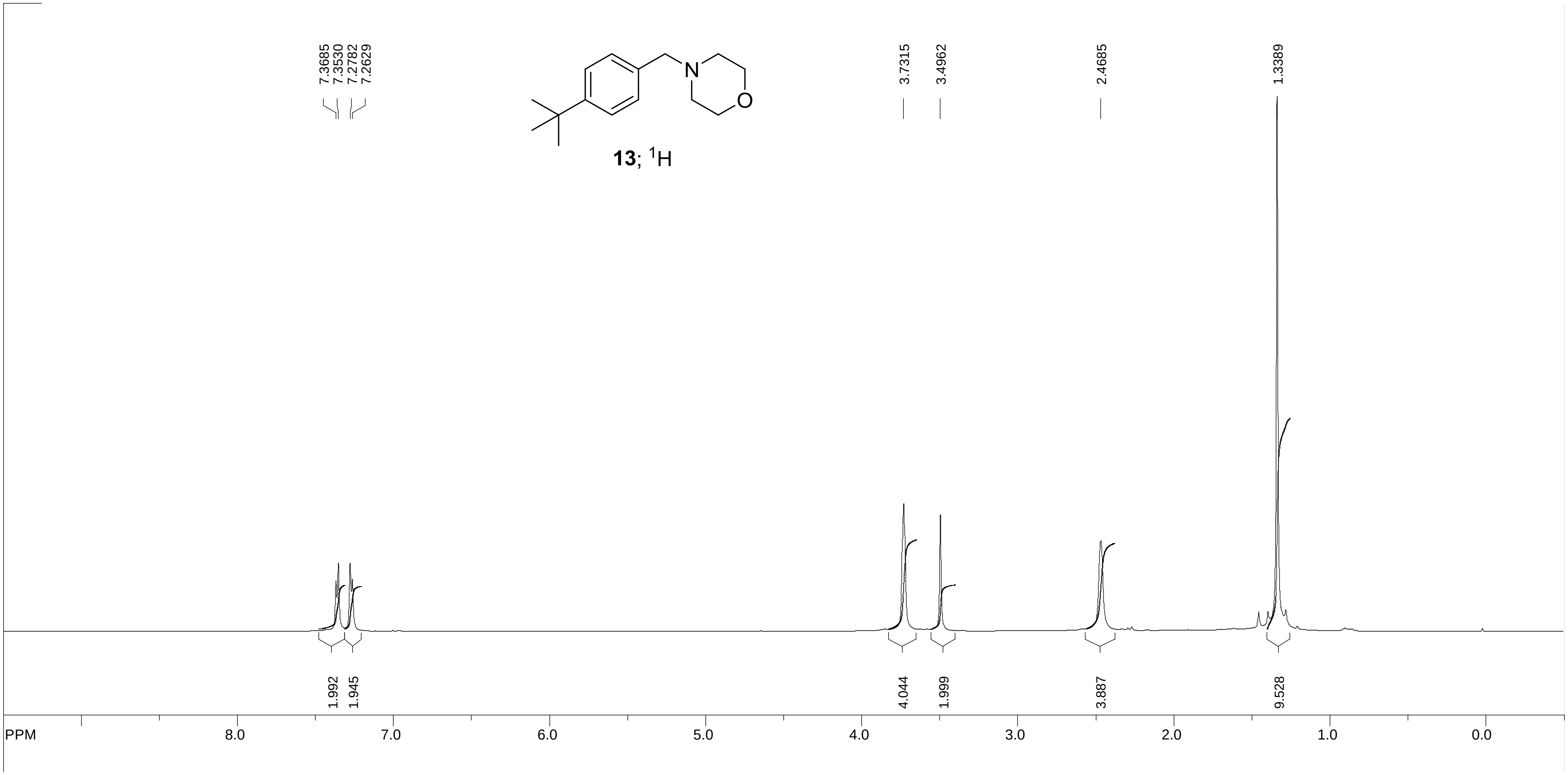

Figure S11: **^{1}H NMR spectrum of compound SM-1.**

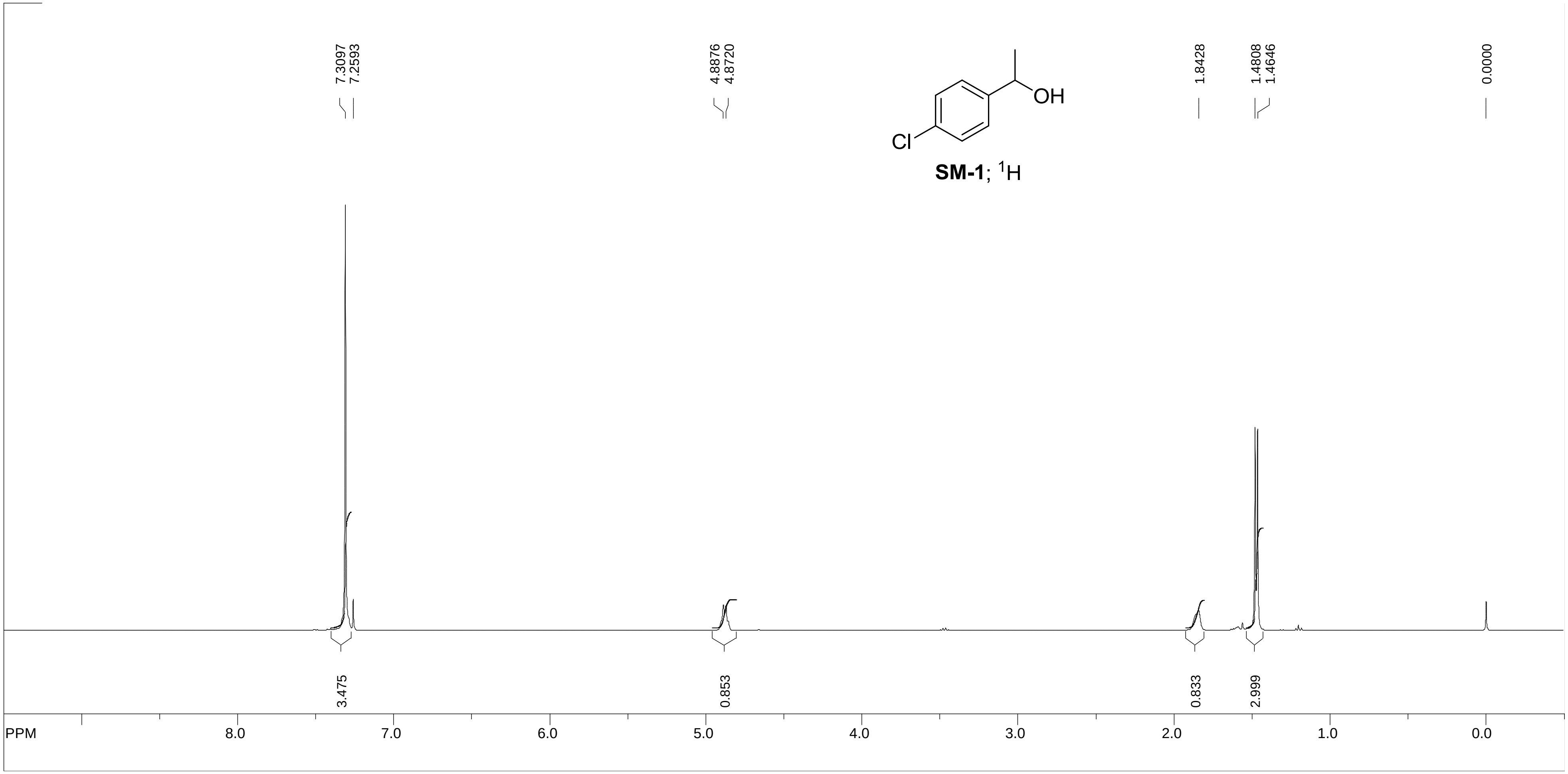

Figure S12: **^{1}H NMR spectrum of compound SM-2.**

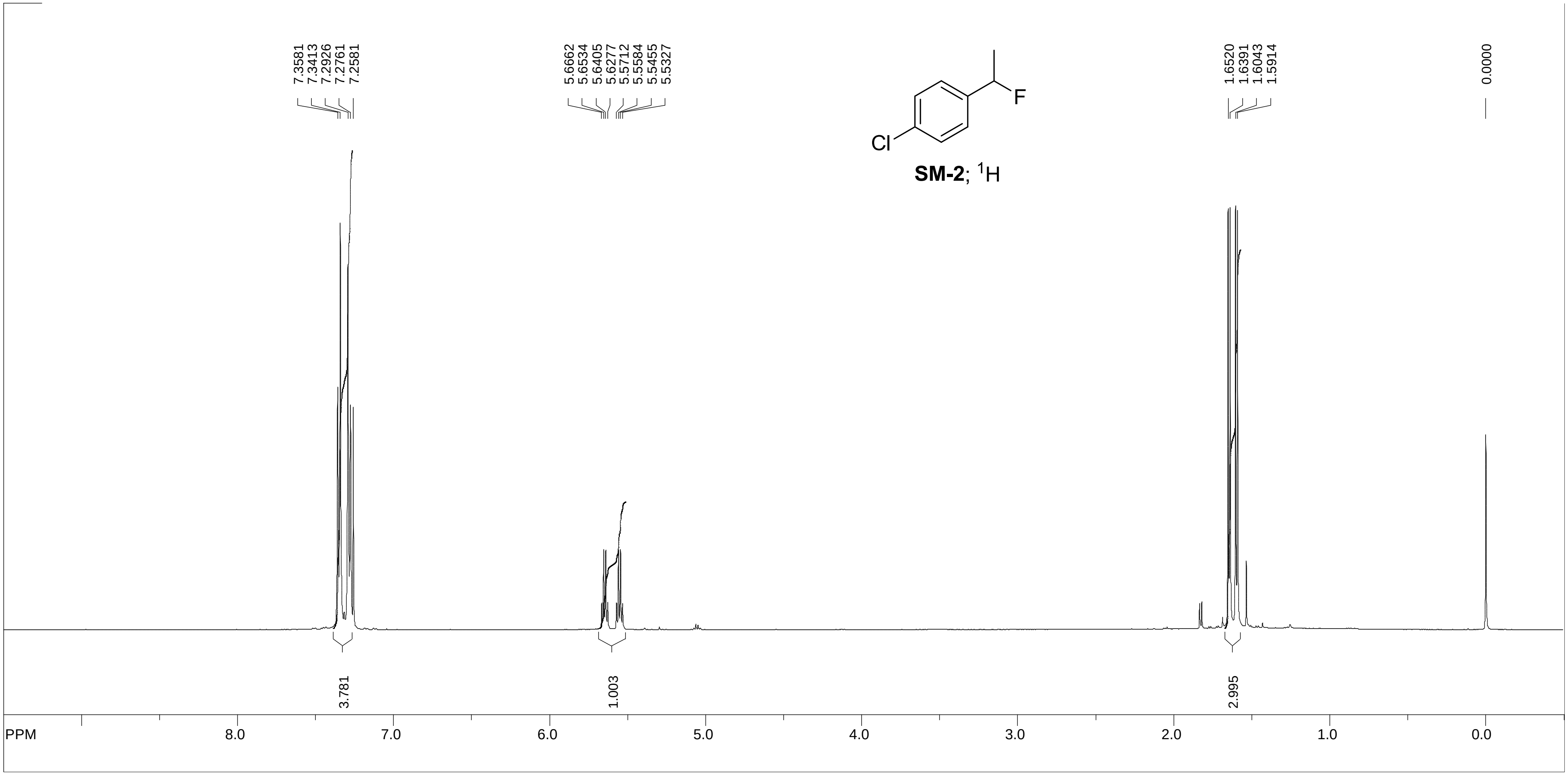

NMR spectra of all new compounds

Figure S13: **^{1}H NMR spectrum of compound 6.**

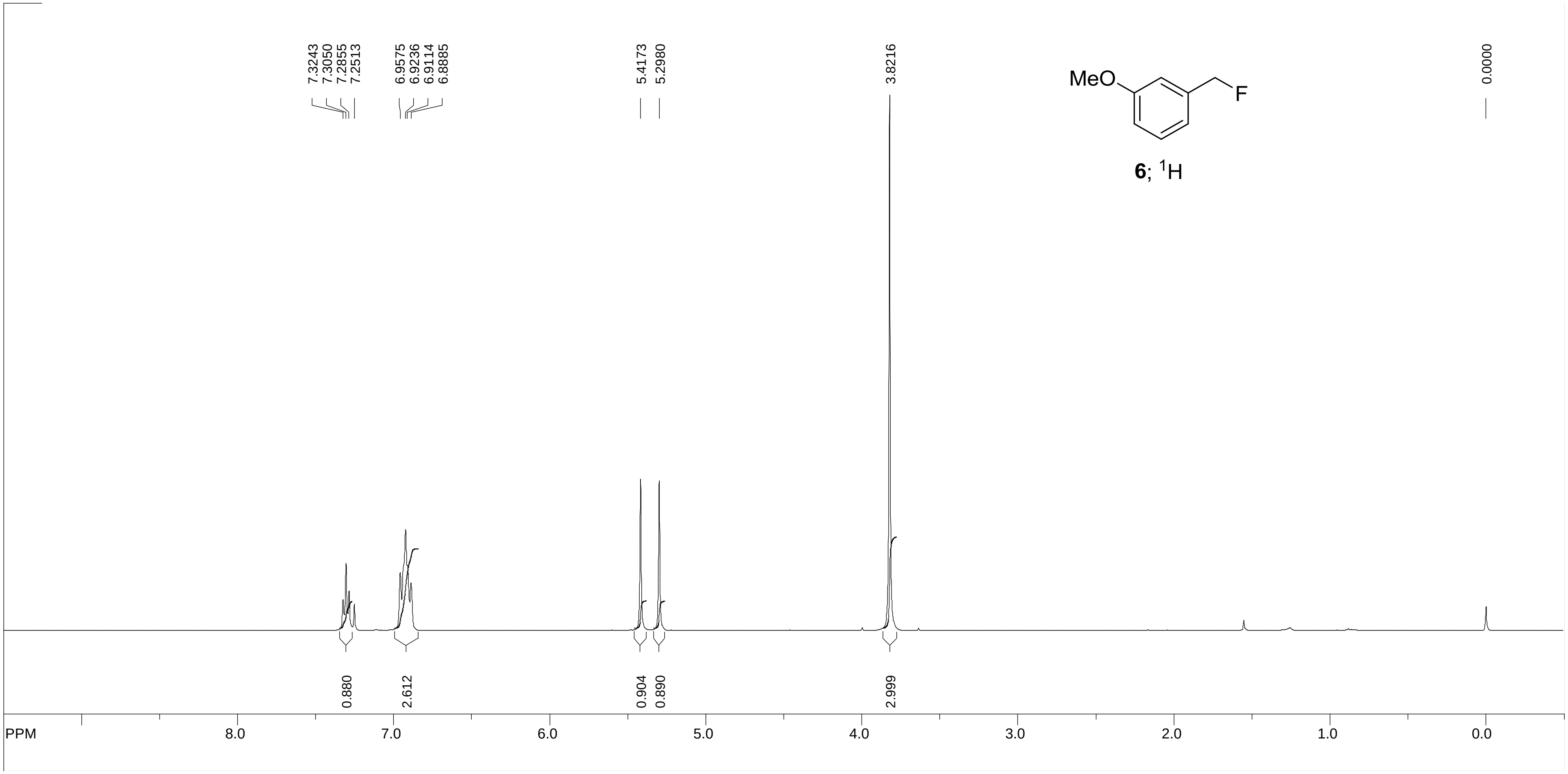

Figure S14: **^{19}F NMR spectrum of compound 6.**

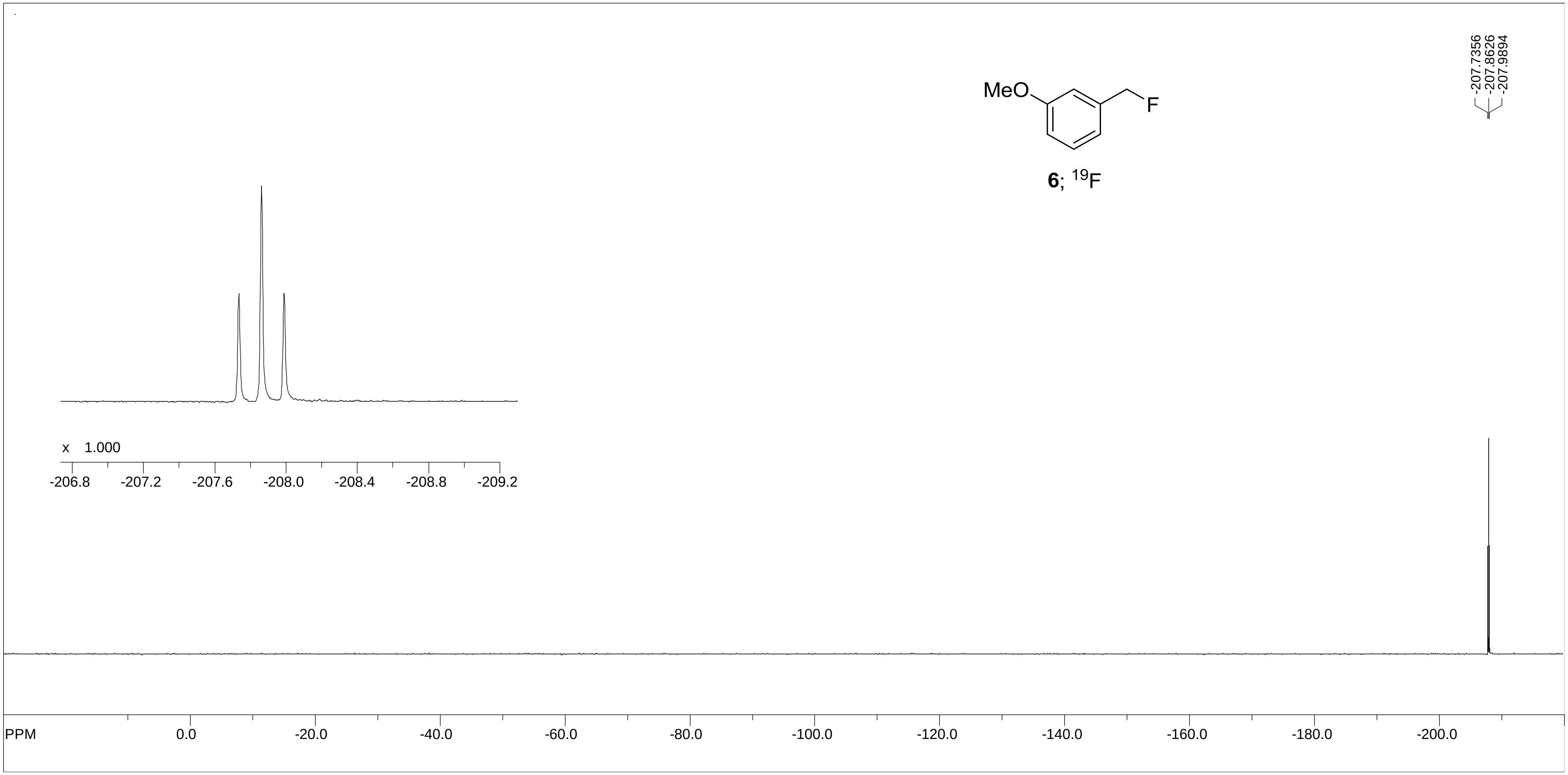

Figure S15: **^{13}C NMR spectrum of compound 6.**

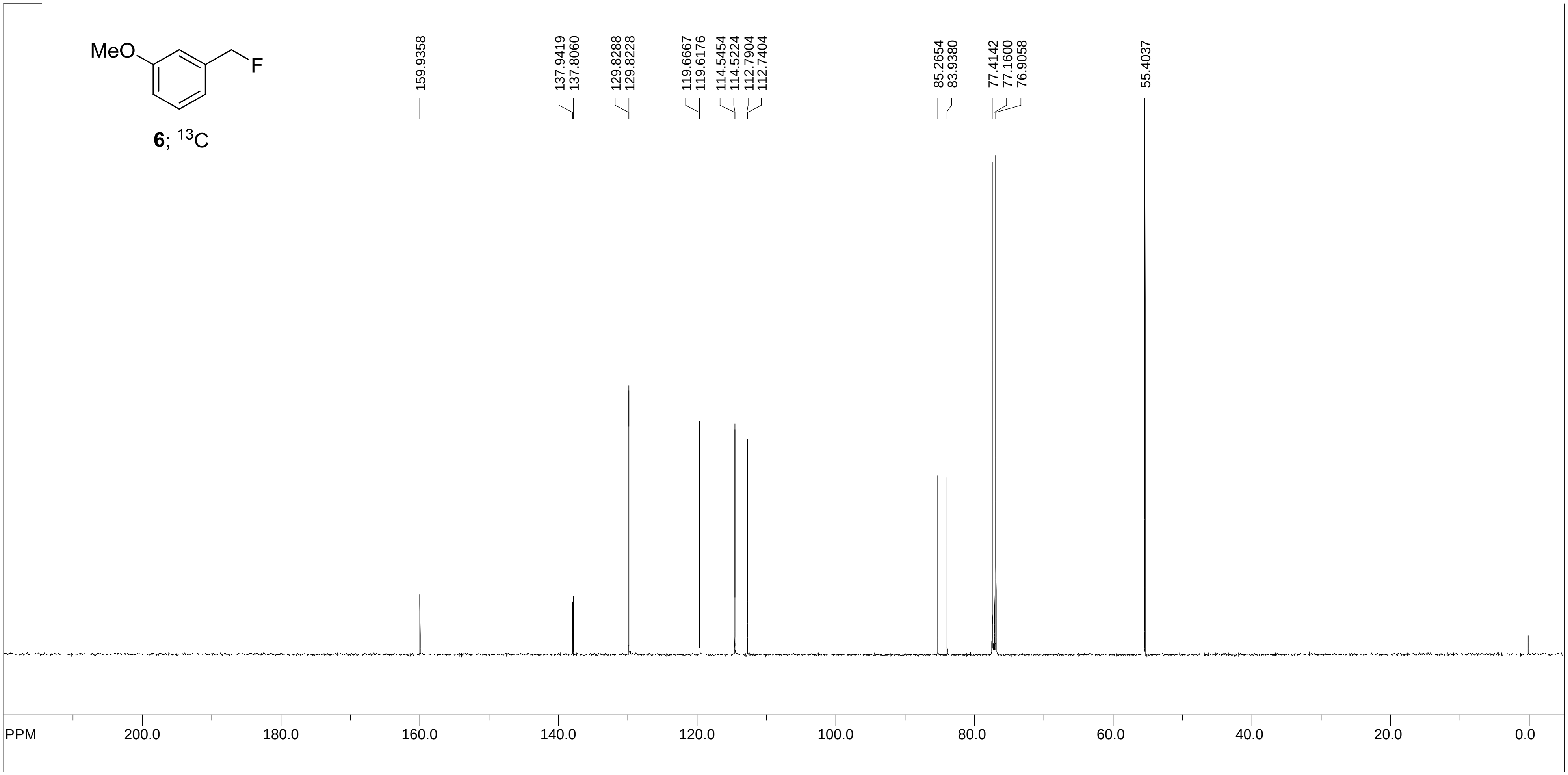

Figure S16: **^{1}H NMR spectrum of compound 9.**

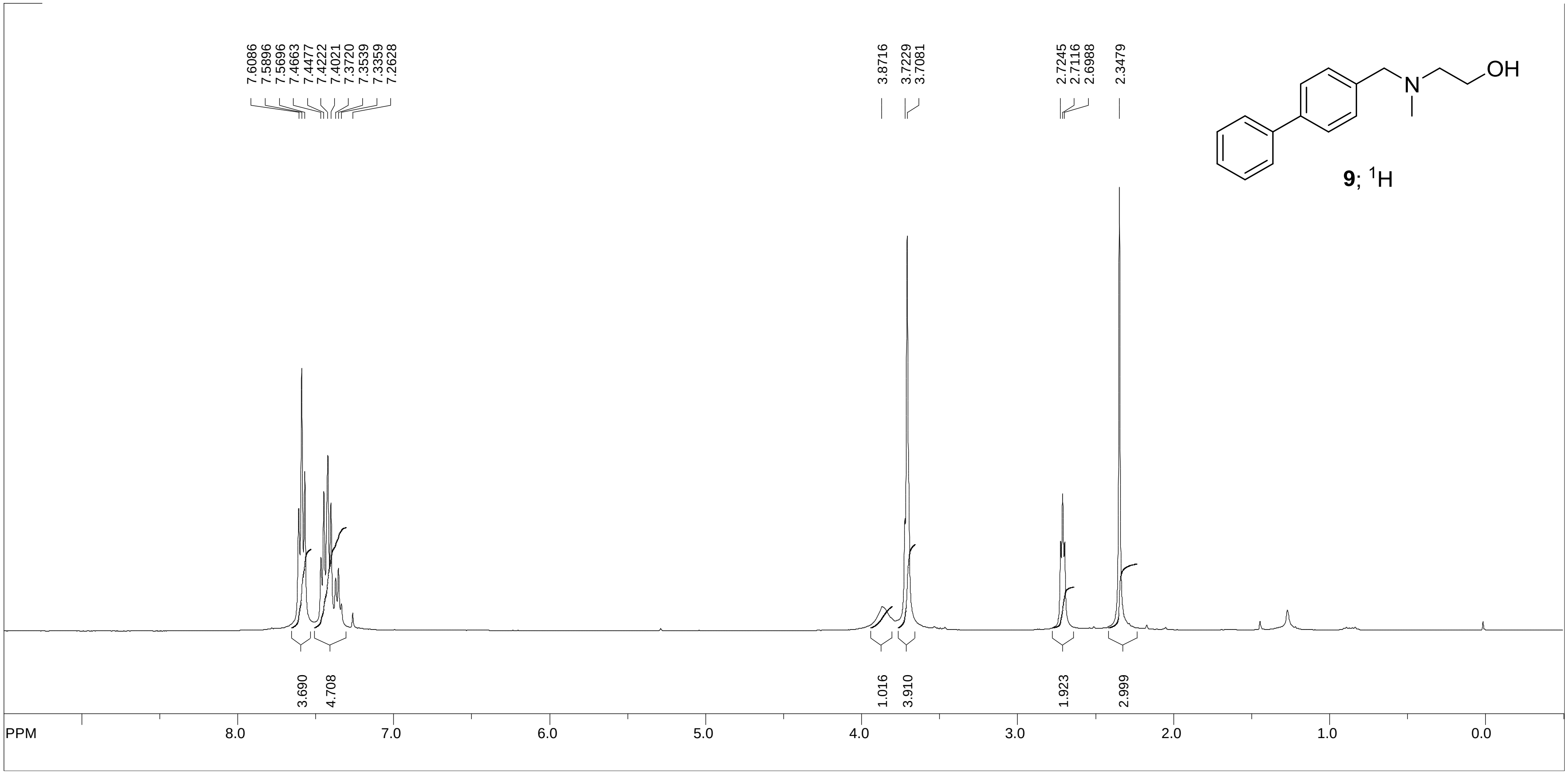

Figure S17: ^{13}C **NMR spectrum of compound 9.**

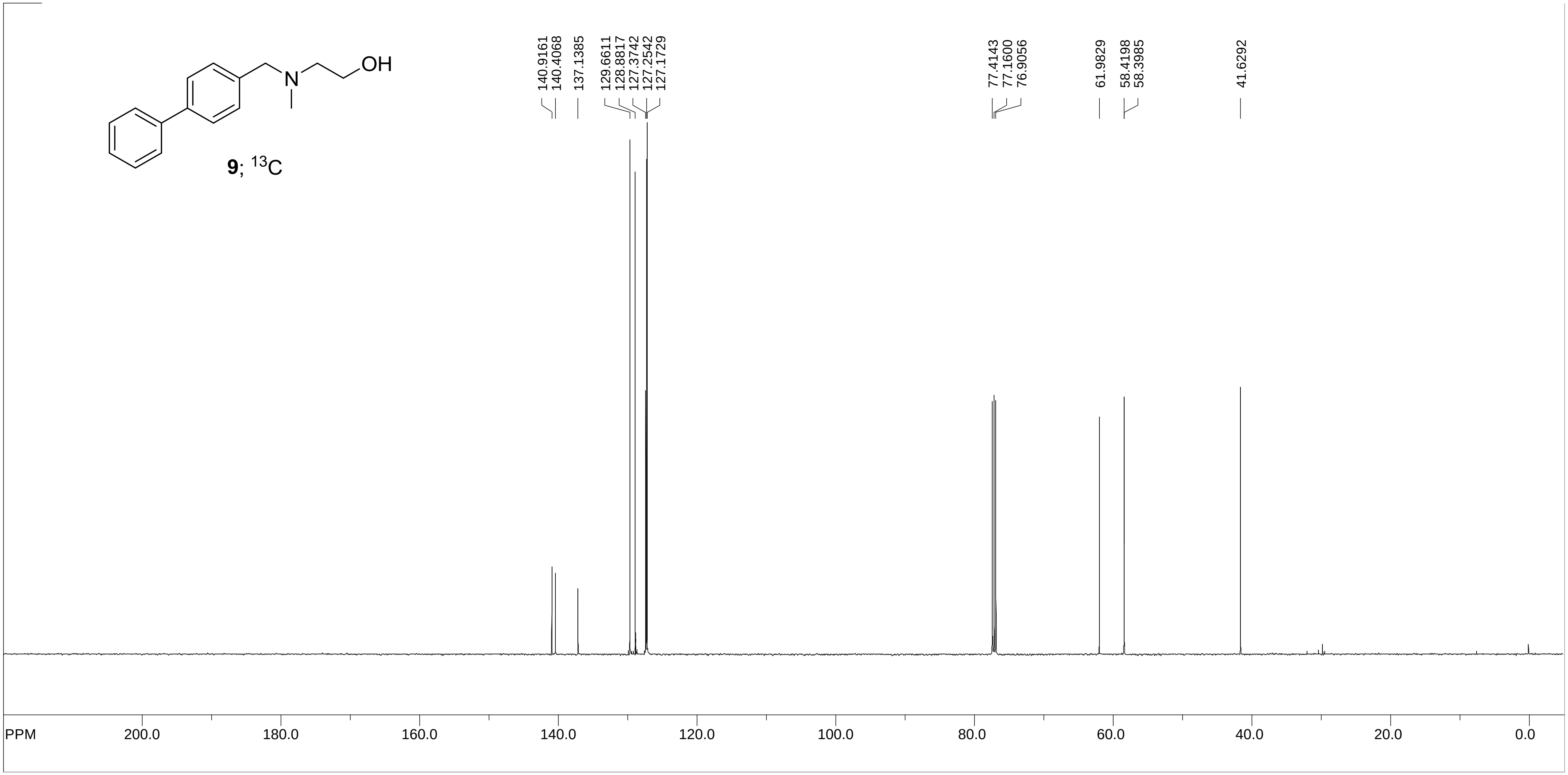

Figure S18: **^{1}H NMR spectrum of compound 14.**

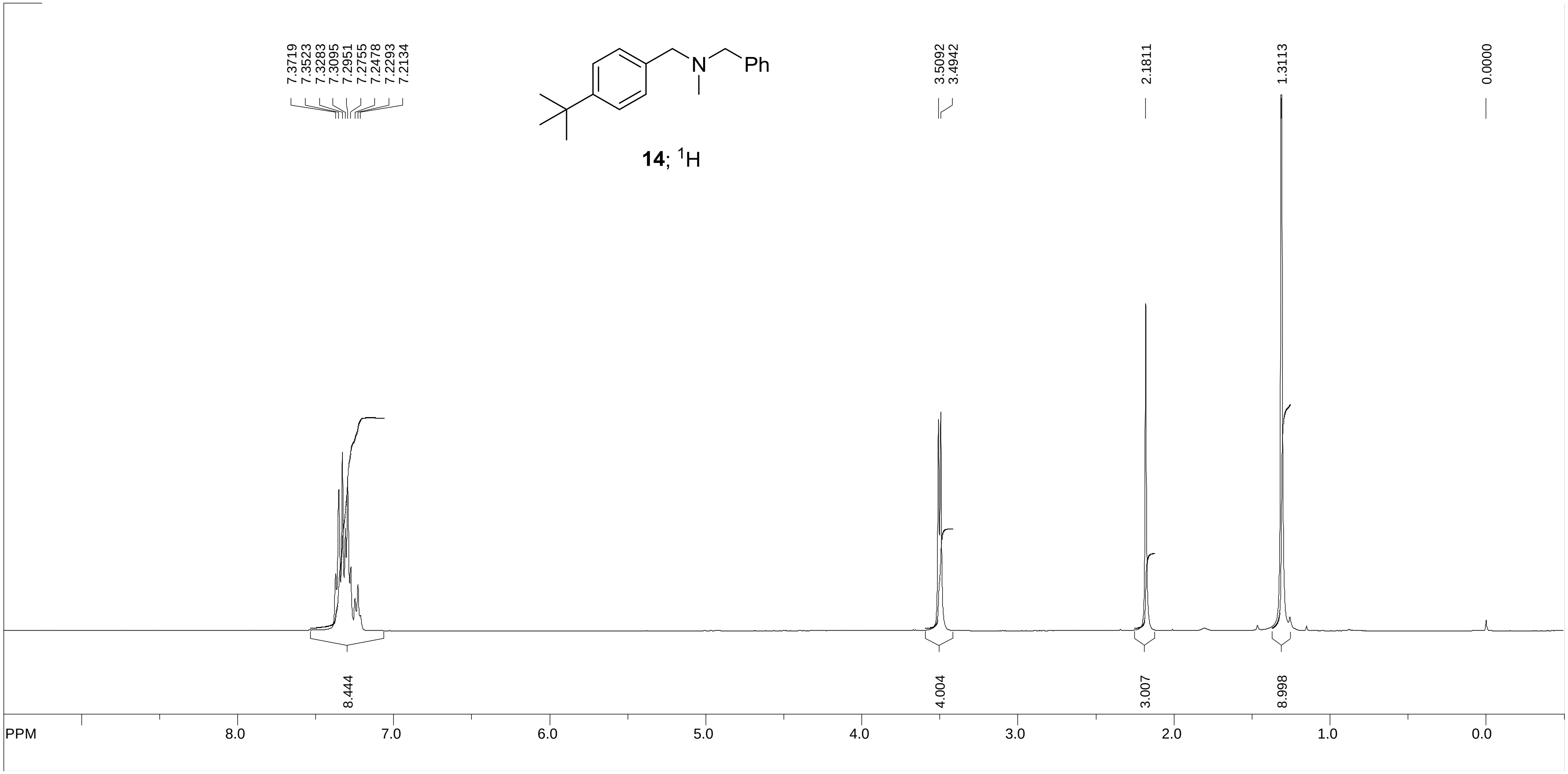

Figure S19: **^{13}C NMR spectrum of compound 14.**

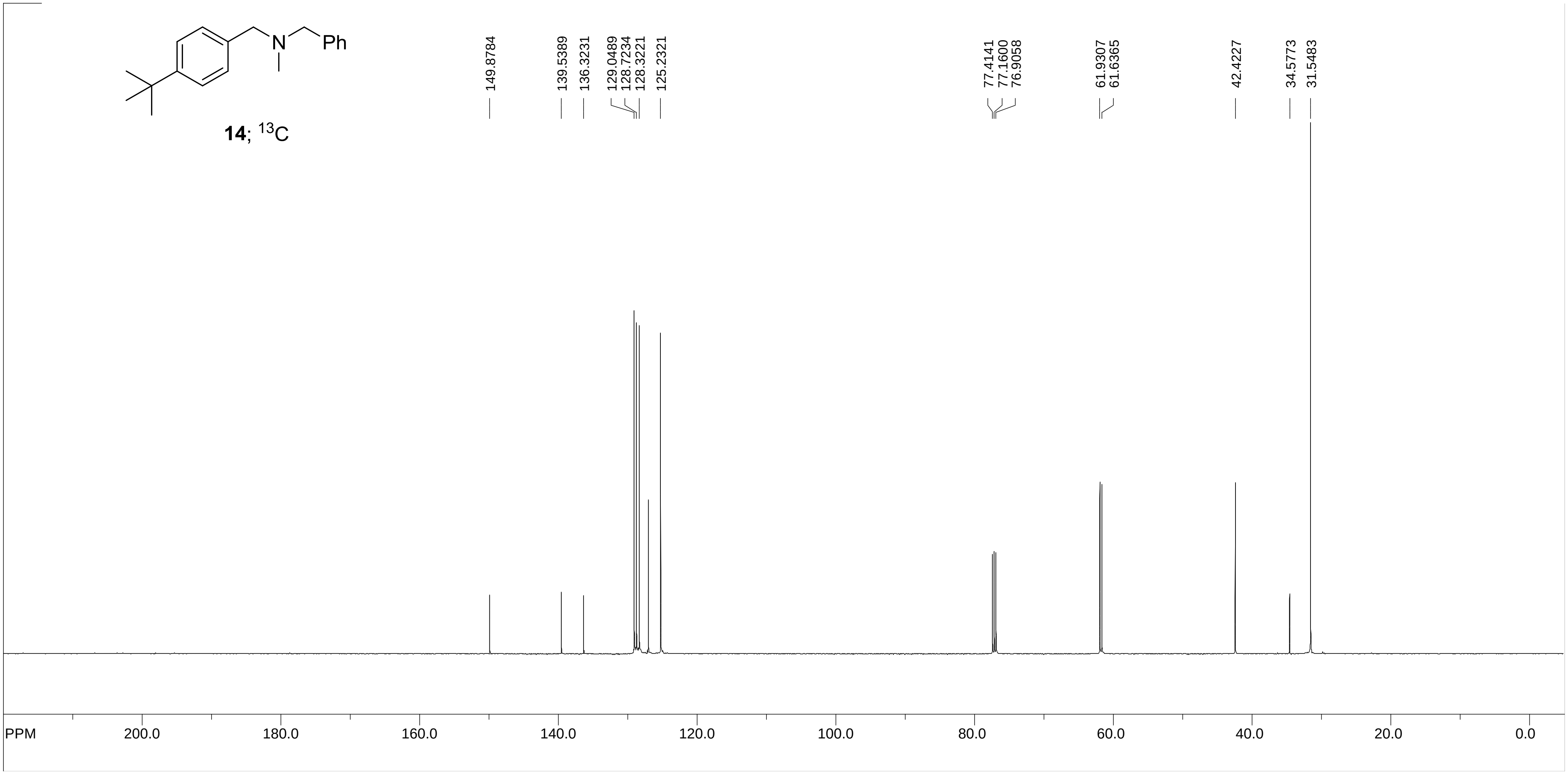

Figure S20: **^{1}H NMR spectrum of compound 15.**

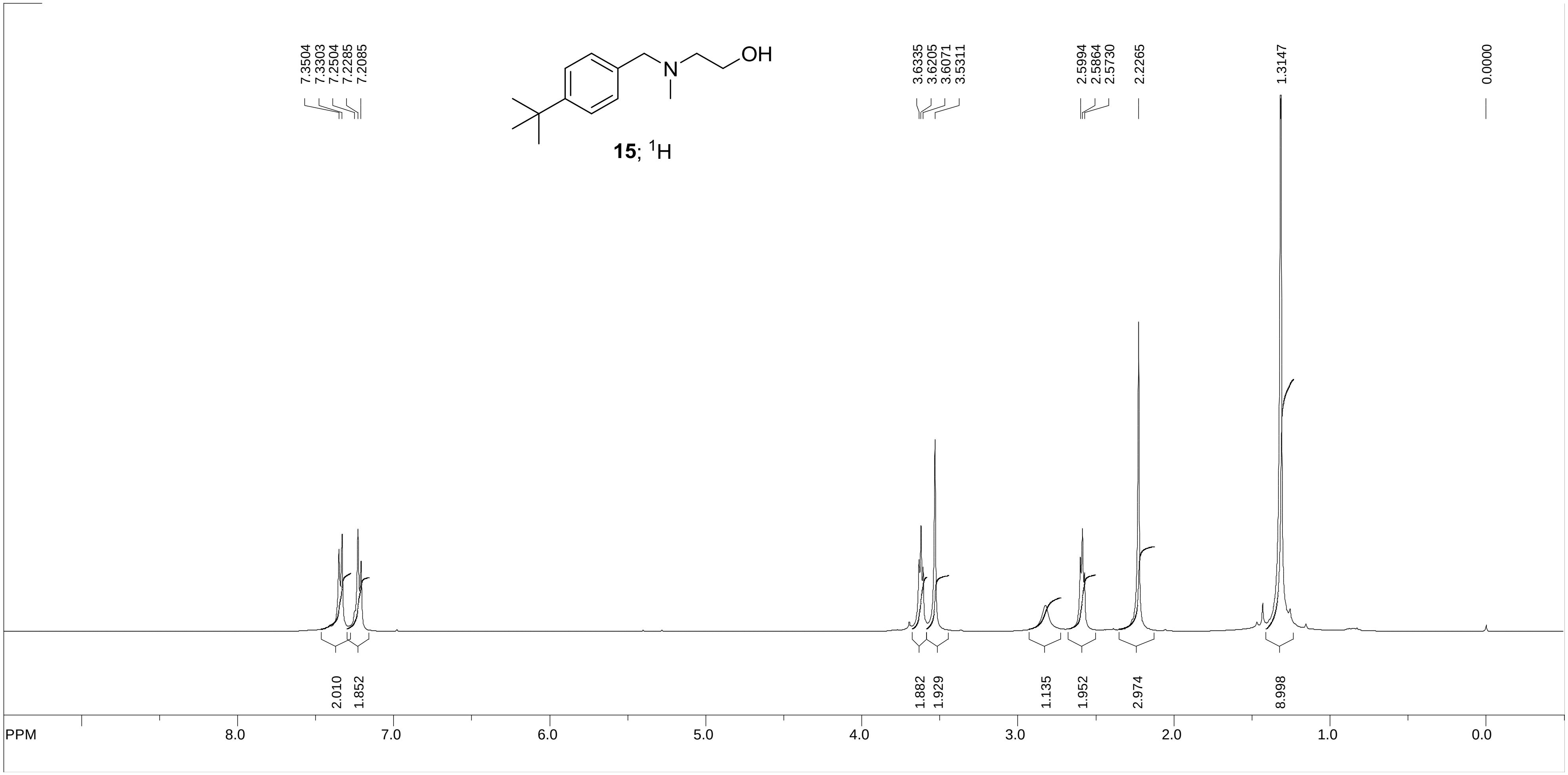

Figure S21: **^{13}C NMR spectrum of compound 15.**

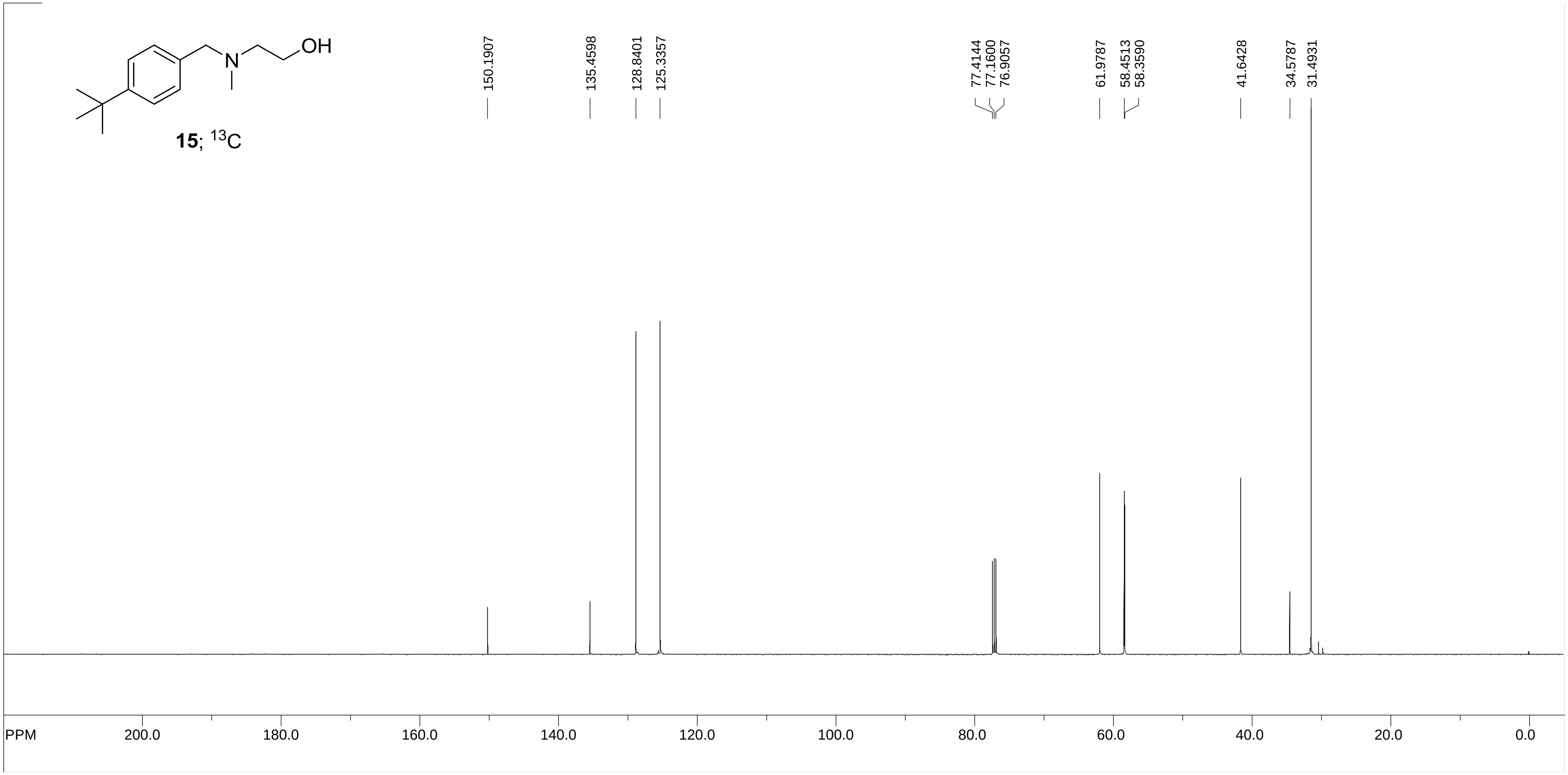

Figure S22: **^{1}H NMR spectrum of compound 16.**

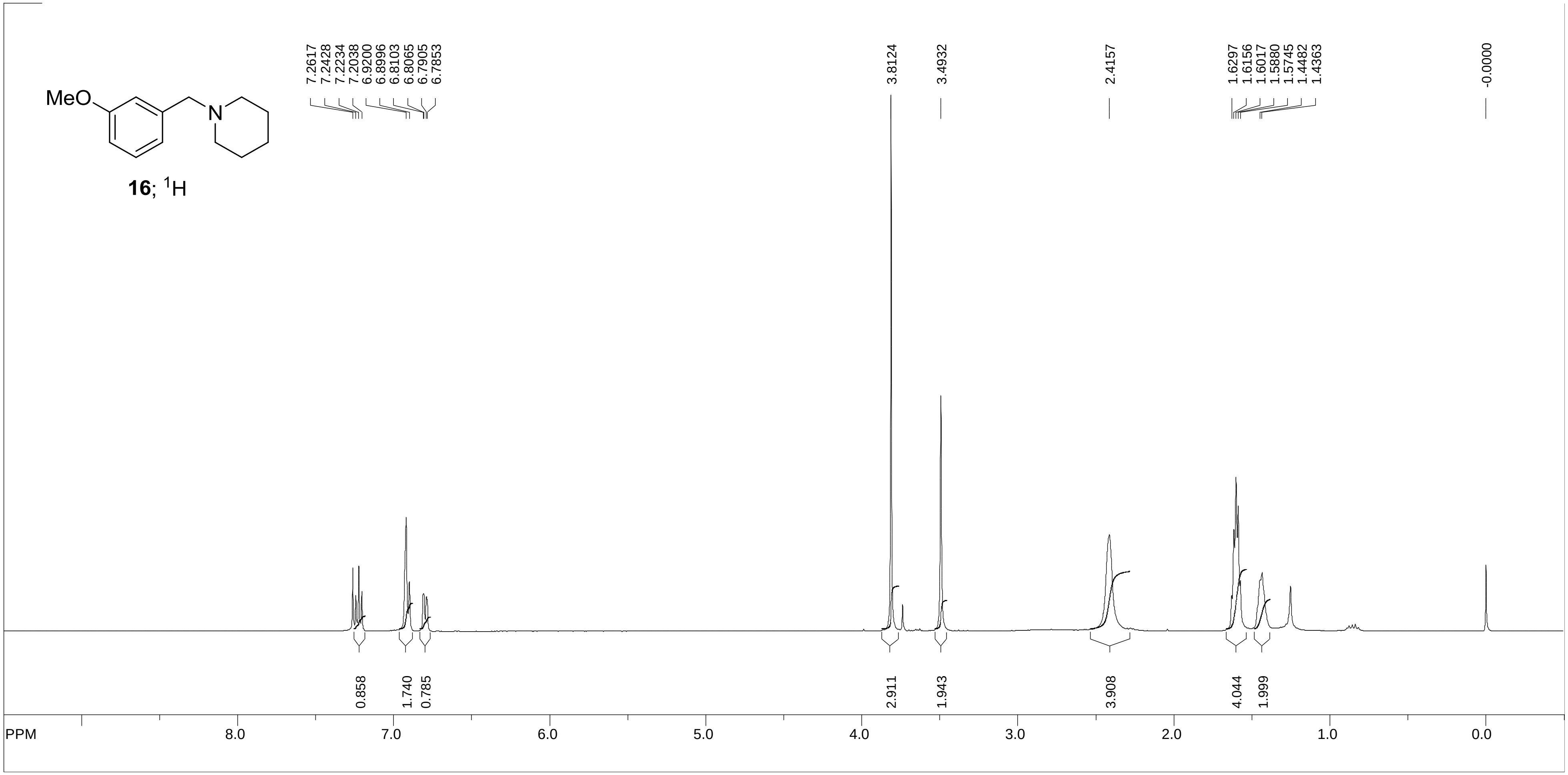

Figure S23: **^{13}C NMR spectrum of compound 16.**

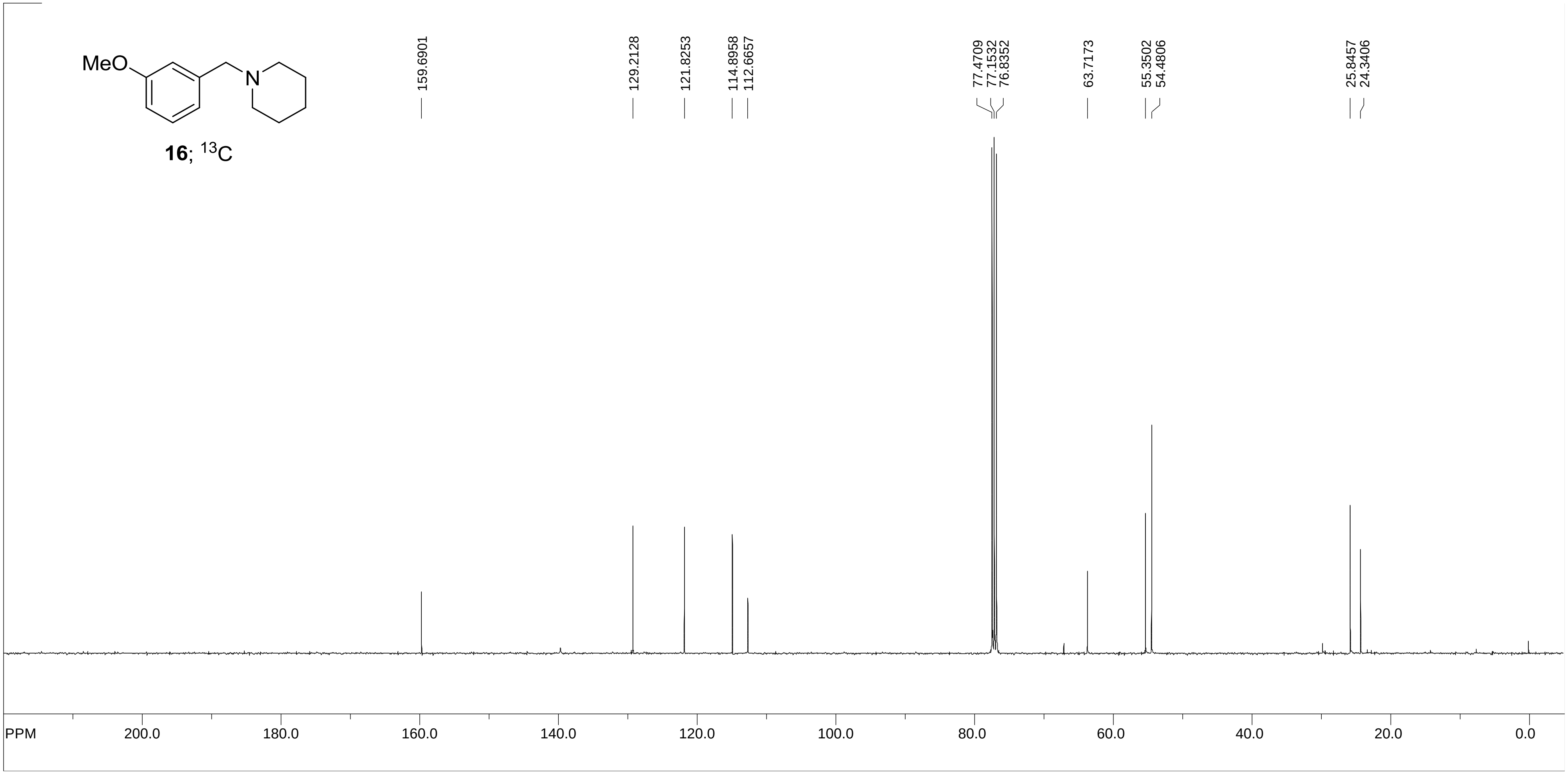

Supplement: File 1 — General methods, synthetic procedures, 1H NMR spectra for known compounds and full characterization of all new compounds. [file Beilstein_J_Org_Chem-09-2451-s001.pdf]
